# Dynamics and regulation of nuclear import and nuclear movements of HIV-1 complexes

**DOI:** 10.1371/journal.ppat.1006570

**Published:** 2017-08-21

**Authors:** Ryan C. Burdick, Krista A. Delviks-Frankenberry, Jianbo Chen, Sanath K. Janaka, Jaya Sastri, Wei-Shau Hu, Vinay K. Pathak

**Affiliations:** 1 Viral Mutation Section, HIV Dynamics and Replication Program, National Cancer Institute at Frederick, Frederick, MD, United States of America; 2 Viral Recombination Section, HIV Dynamics and Replication Program, National Cancer Institute at Frederick, Frederick, MD, United States of America; Fred Hutchinson Cancer Research Center, UNITED STATES

## Abstract

The dynamics and regulation of HIV-1 nuclear import and its intranuclear movements after import have not been studied. To elucidate these essential HIV-1 post-entry events, we labeled viral complexes with two fluorescently tagged virion-incorporated proteins (APOBEC3F or integrase), and analyzed the HIV-1 dynamics of nuclear envelope (NE) docking, nuclear import, and intranuclear movements in living cells. We observed that HIV-1 complexes exhibit unusually long NE residence times (1.5±1.6 hrs) compared to most cellular cargos, which are imported into the nuclei within milliseconds. Furthermore, nuclear import requires HIV-1 capsid (CA) and nuclear pore protein Nup358, and results in significant loss of CA, indicating that one of the viral core uncoating steps occurs during nuclear import. Our results showed that the CA-Cyclophilin A interaction regulates the dynamics of nuclear import by delaying the time of NE docking as well as transport through the nuclear pore, but blocking reverse transcription has no effect on the kinetics of nuclear import. We also visualized the translocation of viral complexes docked at the NE into the nucleus and analyzed their nuclear movements and determined that viral complexes exhibited a brief fast phase (<9 min), followed by a long slow phase lasting several hours. A comparison of the movement of viral complexes to those of proviral transcription sites supports the hypothesis that HIV-1 complexes quickly tether to chromatin at or near their sites of integration in both wild-type cells and cells in which LEDGF/p75 was deleted using CRISPR/cas9, indicating that the tethering interactions do not require LEDGF/p75. These studies provide novel insights into the dynamics of viral complex-NE association, regulation of nuclear import, viral core uncoating, and intranuclear movements that precede integration site selection.

## Author summary

Although nuclear import of HIV-1 is essential for viral replication, many aspects of this process are currently unknown. Here, we defined the dynamics of HIV-1 nuclear envelope (NE) docking, nuclear import and its relationship to viral core uncoating, and intranuclear movements. We observed that HIV-1 complexes exhibit an unusually long residence time at the NE (∼1.5 hrs) compared to other cellular and viral cargos, and that HIV-1 capsid (CA) and host nuclear pore protein Nup358 are required for NE docking and nuclear import. Soon after import, the viral complexes exhibit a brief fast phase of movement, followed by a long slow phase, during which their movement is similar to that of integrated proviruses, suggesting that they rapidly become tethered to chromatin through interactions that do not require LEDGF/p75. Importantly, we found that NE association and nuclear import is regulated by the CA-cyclophilin A interaction, but not reverse transcription, and that one of the viral core uncoating steps, characterized by substantial loss of CA, occurs concurrently with nuclear import.

## Introduction

HIV-1 enters and travels through the cytoplasm of an infected cell, reverse transcribes its genomic RNA into double-stranded DNA and forms a preintegration complex (PIC), crosses through a nuclear pore, and integrates its DNA into the host genome (reviewed in [1]). Although movement of fluorescently labeled HIV-1 complexes in infected living cells has been described [2–4], the dynamics with which individual HIV-1 complexes encounter nuclear pores and are imported into the nucleus, as well as the molecular events that regulate these dynamics, are not well understood, largely because these events have not been extensively studied in living cells.

HIV-1 viral cores are large (61-nm width, 120-nm length) compared to the 40-nm size limit for translocation through a nuclear pore complex (NPC) [5,6]; thus, it is generally assumed that viral complexes must be disassembled before nuclear import can take place (reviewed in [7]). If viral complexes are disassembled in the cytoplasm and are converted to a form/size that is competent for nuclear import, then one might expect that the viral complexes exhibit a very short residence time at the nuclear envelope (NE). This would be a scenario that is similar to adeno-associated virus 2 complexes, which are 25-nm diameter in size, and other large cellular cargos, which dock at the NE and are transported through the nuclear pore within milliseconds [8–10]. On the other hand, if the viral complexes undergo capsid disassembly at the NE then they would be expected to reside at the NE for a long time prior to import, during which uncoating occurs to generate a viral complex that can be translocated through a nuclear pore. A third possibility is that uncoating can occur either in the cytoplasm or at the NE; in this scenario, one would expect that the length of time a viral complex is in the cytoplasm would be inversely correlated with their residence time at the NE. Thus, live-cell imaging analysis of the length of time viral complexes reside in the cytoplasm and at the NE can provide valuable insight into not only the process of nuclear import, but also the process of viral core uncoating, and facilitate the identification of viral and host factors that regulate these events.

Currently, the translocation of viral complexes into the nucleus and their nuclear movements after import have not been observed. Understanding these processes can provide insights into the dynamics and molecular interactions of viral complexes with chromatin or other macromolecules that precede integration site selection and provirus formation. Although currently there is a debate as to whether HIV-1 integrates into genes near the periphery of the nucleus [11,12] or in genes throughout the nucleus [13], the kinetics of association of viral complexes with chromatin prior to integration, and viral and host factors that may be essential for tethering viral complexes to chromatin, have not been investigated. Analysis of the intranuclear movement of viral complexes can provide insights into the extent and the kinetics with which viral complexes can freely diffuse in the nucleus, whether they form multiple transient contacts with the chromatin before selecting the site of integration, and whether they stably associate with specific sites on chromatin before integration. The intranuclear location of viral complexes can also provide information regarding the nuclear location of integration sites relative to the nuclear point of entry. Lastly, we previously observed that only 1 in 50 viral complexes in the cytoplasm are imported into the nucleus, which is similar to the number of proviruses that integrated and expressed a reporter gene [14]; thus, visualizing the nuclear import of viral complexes can help to identify and study the complexes that are likely to complete viral replication away from the vast majority of the cytoplasmic viral complexes that do not complete viral replication.

APOBEC3F (A3F) and APOBEC3G (A3G) are host restriction factors that are incorporated into virions in virus producing cells and potently inhibit virus replication [15–19]. We [20,21] and others [22] recently demonstrated that A3F and A3G inhibited viral integration and that A3F has a higher affinity for double-stranded DNA than A3G, suggesting that A3F and A3G might stay associated with the viral complex in the nucleus. Subsequently, we produced virions labeled with yellow fluorescent protein (YFP)-tagged A3F (A3F-YFP) or A3G (A3G-YFP), infected cells, and then analyzed their association with viral complexes. We found that A3F-YFP, and to a lesser extent A3G-YFP, remained stably associated with viral complexes and could be used to visualize viral complexes in the nuclei of infected cells [14]. We also found that A3F-YFP colocalized with viral nucleic acid in the nuclei of infected cells, indicating that A3F-YFP indeed remains stably associated with nuclear viral complexes. Some recent studies have shown that a Vpr-integrase-green fluorescent protein fusion protein (Vpr-IN-GFP) could be used to label viral complexes [23,24]; Vpr-IN-GFP is incorporated into virions, and proteolytic processing between Vpr and IN-GFP by viral protease during virion maturation results in the release of IN-GFP, which can be used to detect nuclear HIV-1 complexes.

Cyclophilin A (CypA) is a host peptidylprolyl isomerase that binds to an exposed loop on the surface of CA and is incorporated into virions [25–27]. The CA-CypA interaction can be disrupted by treatment with cyclosporine A (CsA), which binds to CypA, or by introducing mutations into the CA loop that is involved in binding to CypA (reviewed in [7]). Disruption of the CA-CypA interaction results in a reduction in viral titers in primary CD4^+^ T cells, monocyte derived macrophages, and in some T cell lines, but has minimal effects on the viral infectivity in other cell lines such as HeLa cells [28–31]. It is also known that disruption of the CA-CypA interaction influences nuclear import and the requirement for some nuclear pore proteins [32–35]. However, the influence of CypA on the timing and regulation of HIV-1 nuclear import has not been studied.

The detailed dynamics of HIV-1 association with the NE and their import into the nucleus in living cells have not been previously analyzed. Arhel and colleagues [2], and more recently Dharan and colleagues [36], observed the docking of a few HIV-1 complexes with the NE in living cells, but they did not determine the functional relevance or the kinetics of these associations. We and others have observed association of viral complexes with the NE in fixed cells; however, these studies cannot provide insights into the kinetics and stability of NE association [3,14,37–42].

Here, we analyzed living cells infected with HIV-1 particles labeled with either A3F-YFP or Vpr-integrase-YFP fusion protein (hereafter referred to as IN-YFP), and characterized the dynamics with which HIV-1 complexes stably associate with the NE, translocate from the cytoplasm into the nucleus, move after nuclear entry, and the viral and host factors that regulate these events. Our studies indicate that long residence times at the NE involving CA and host nuclear pore protein Nup358 are required for nuclear import and that the timing of nuclear import is regulated by CA-CypA interactions, but not reverse transcription. Additionally, the nuclear import of viral complexes is correlated with substantial loss of CA, indicating that at least a portion of the viral core uncoating occurs during nuclear import. After nuclear entry, the viral complexes exhibited a brief fast phase during which the viral complexes may move away from the NE, followed by a long slow phase during which the viral complexes move at a rate similar to integrated proviruses, supporting the hypothesis that the viral complexes are tethered to chromatin and/or other large macromolecules and remain at or near their future site of integration.

## Results

### Translocation of viral complexes from the NE to the nucleus is associated with substantial loss of CA

**Fig 1:**
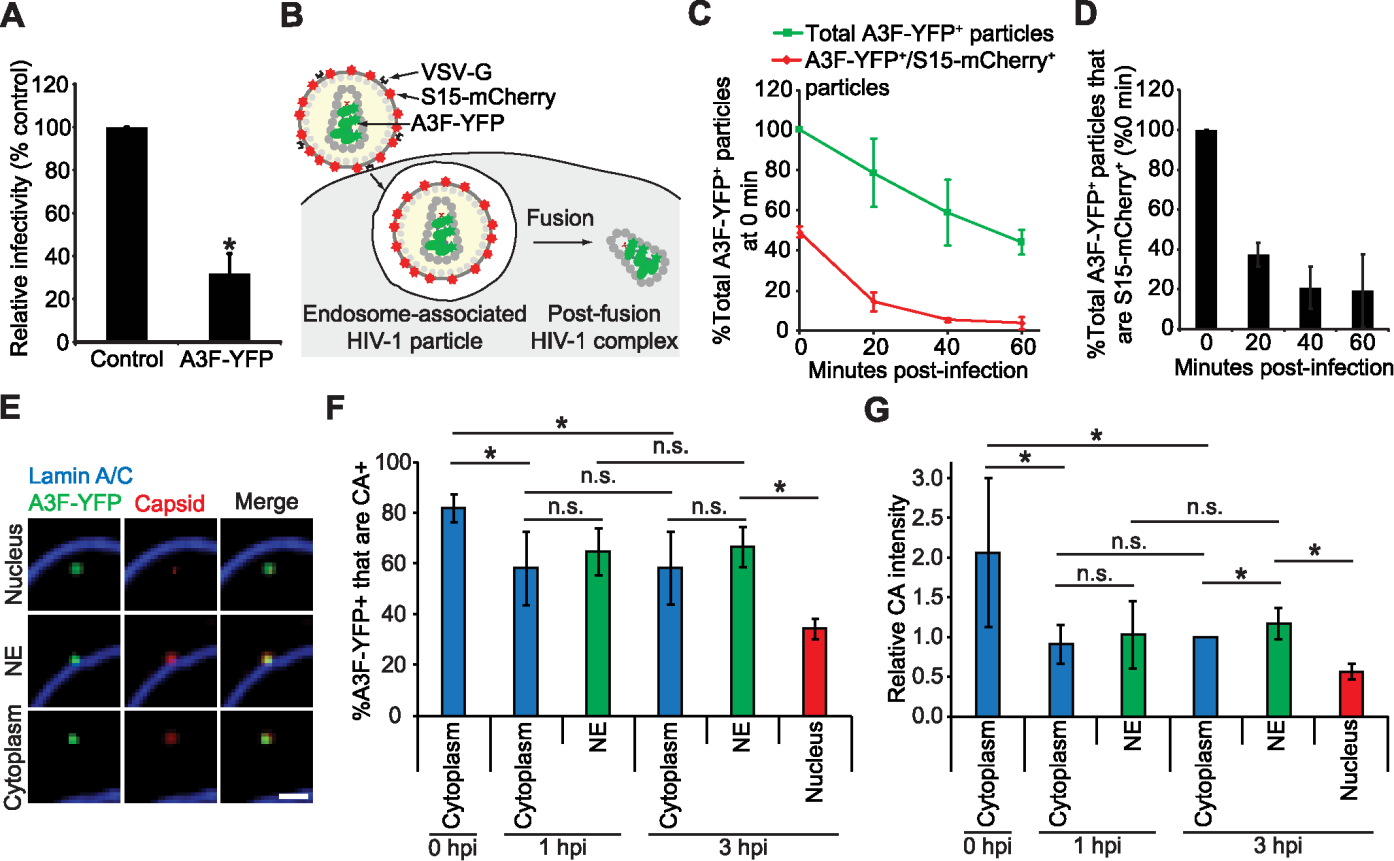
Virus infectivity, fusion kinetics, and characterization of A3F-YFP complexes in infected cells. **(A)** Relative infectivity of an HIV-1 GFP-reporter virus without any label (Control) or with A3F-YFP. The percentage of GFP^+^ cells was determined by flow cytometry 48 hrs after infection. (**B-D)** Determination of fusion efficiency. HeLa cells were infected with VSV-G Env-pseudotyped HIV-1 labeled with A3F-YFP (green) and S15-mCherry (red) membrane marker. An endosome-associated HIV-1 complex (YFP^+^/mCherry^+^) and a post-fusion HIV-1 complex (YFP^+^/mCherry^–^) are shown in **(B)**. **(C)** The percentage of total A3F-YFP^+^ and A3F-YFP^+^/S15-mCherry^+^ dual-labeled particles relative to the total A3F-YFP^+^ particles at the time of infection (set to 100%). The S15-mCherry labeling efficiency of the virions used for infections was ∼50%. **(D)** The ratio of A3F-YFP^+^/S15-mCherry^+^ dual-labeled particles to total A3F-YFP labeled particles was determined for each time point, and the ratio at the 0-min post-infection time point was set to 100% to account for the initial S15-mCherry labeling efficiency. The relative proportion of A3F-YFP^+^/S15-mCherry^+^ dual labeled particles remaining at each time point are plotted. For **(C-D)**, error bars indicate standard deviations (SD) of three experiments; an average of ∼1425 YFP particles from an average of ∼137 cells were analyzed per time point for each experiment. **(E-G)** Characterization of A3F-YFP complexes in infected cells. HeLa cells were infected with VSV-G pseudotyped HIV-1 labeled with A3F-YFP and fixed at 0, 1, and 3 hrs post-infection. Examples of A3F-YFP (green) complexes that co-localize with CA stained with anti-CA antibody (red) in the nucleus (top panels), at the nuclear envelope (NE; middle panels), and in the cytoplasm (bottom panels) are shown in **(E)**. The NE was stained blue with anti-Lamin A/C antibody. Scale bar, 2 μm. **(F)** The percentage of A3F-YFP^+^ signals that are CA^+^ are shown at 0, 1 and 3 hours post-infection (hpi) for viral complexes in the cytoplasm (all time points), viral complexes associated with the NE (1- and 3-hr time points), and viral complexes in the nucleus (3 hr time point). **(G)** The average CA signal intensity associated with A3F-YFP signals in the cytoplasm for the 3 hr time point was set to 1, and the relative CA signal intensities are shown for particles in the cytoplasm, particles associated with the NE, and particles in the nucleus. For **(F-G)**, error bars indicate SD of 4 experiments; the particles from an average of ∼100 cells were analyzed for each experiment; the total A3F-YFP particles analyzed were 6028–7937 in the cytoplasm, 1656–1690 at the NE, and 1152 in the nucleus. On average, the CA signals were ∼10-fold higher than background signals. *, *P* ≤ 0.05; n.s., not significant (*P* > 0.05), *t*-test.

To visualize viral complexes in infected cells, we labeled HIV-1 virions with A3F-YFP that were pseudotyped with vesicular stomatitis virus G protein (VSV-G). VSV-G pseudotyped virions enter cells through endocytosis, and fusion of the viral and endosomal membranes leads to the release of the viral core into the cytoplasm (reviewed in [43]). We determined that the infectivity of the virions containing A3F-YFP was modestly reduced by threefold compared to control virions without any label (Fig 1A). To ensure that we visualized the movement of viral complexes after fusion and not those trapped in endosomes, we determined the efficiency and kinetics of fusion. For these experiments, the virus particles were labeled with A3F-YFP, which is incorporated into the virion core, as well as S15-mCherry; S15-mCherry is a protein that is non-specifically incorporated into membranes, and serves as a marker that can be used to distinguish post-fusion viral complexes (S15-mCherry^–^) from those that remain in endosomes (S15-mCherry^+^) (Fig 1B) [44]. We determined the proportion of all A3F-YFP labeled virions that remained 20, 40, and 60 minutes after infection relative to the number of virions at the time of infection (0-min time point, which was set to 100%; Fig 1C). There was progressive loss of A3F-YFP labeled virions, and ∼44% of the A3F-YFP labeled virions remained 60-min after infection. A3F-YFP labeled virions may be degraded after endocytosis or the YFP signal may be lost due to diffusion of A3F-YFP away from some viral complexes, such as immature virions with incompletely closed viral cores. Approximately 50% of the A3F-YFP labeled virions were also labeled with S15-mCherry at the time of infection and, at the 60-min time point, the proportion of dual-labeled virions was reduced to ∼9% of the total A3F-YFP labeled particles at the time of infection. The efficiency of fusion was determined by dividing the number of dual-labeled particles by the total A3F-YFP labeled particles at each time point, and setting the ratio at the 0-min time point to 100% (Fig 1D). The percentage of dual-labeled particles remaining relative to the 0-min time point was reduced to ∼20% at the 40- and 60-min time points, providing a fusion efficiency of ∼80%, which is consistent with previous reports [44,45]. Based on the observed timing of fusion events, we initiated most live-cell microscopy experiments ∼45 min after infection so that we were primarily visualizing post-fusion viral complexes; because these experiments were initiated after most viruses had fused their membranes with endosomal membranes, virions for these experiments were made without S15-mCherry.

To verify that A3F-YFP signals in infected cells were viral complexes, we fixed infected cells at 0, 1, and 3 hrs post-infection (hpi) and detected HIV-1 capsid (CA) by immunofluorescence staining and confocal microscopy (Fig 1E). Approximately 80% of the A3F-YFP signals in the cytoplasm at the 0 hpi were colocalized with detectable levels of CA, verifying that most of the YFP signals in the cytoplasm were viral complexes (Fig 1F). At 1 and 3 hpi, ∼60% of the viral complexes in the cytoplasm and at the NE were colocalized with CA, which was significantly different from the 0-hr time point, but not significantly different from each other. In contrast, only ∼35% of the YFP signals in the nuclei at the 3-hr time point were associated with CA (Fig 1F). The proportions of CA^+^ viral complexes at the 1-hr time point were not determined because very few nuclear viral complexes were detected at this early time point. We also found that the average relative intensity of the CA signals at the 1-hr time point in the cytoplasm and at the NE were ∼50% of the average CA signal intensities at the 0-hr time point (Fig 1G). Since only ∼1500–2500 of the ∼5000 CA molecules that are packaged into virions form the viral core [5,46], we expect that ∼50 to 70% of the CA protein in virions are monomers that will diffuse away from the viral complexes upon fusion of the viral and host membranes. In addition, the loss of CA signal at the 1-hr time point could be due to loss of CA from the viral cores due to uncoating. We did not observe any significant loss of average CA signal intensity from viral complexes in the cytoplasm or at the NE between 1-hr and 3-hr time points. However, we observed 2.5-fold lower CA signal intensity associated with nuclear complexes compared to NE-associated complexes, indicating that a significant amount of CA is lost upon nuclear entry (Fig 1G).

### Kinetics of NE association of viral complexes

**Fig 2:**
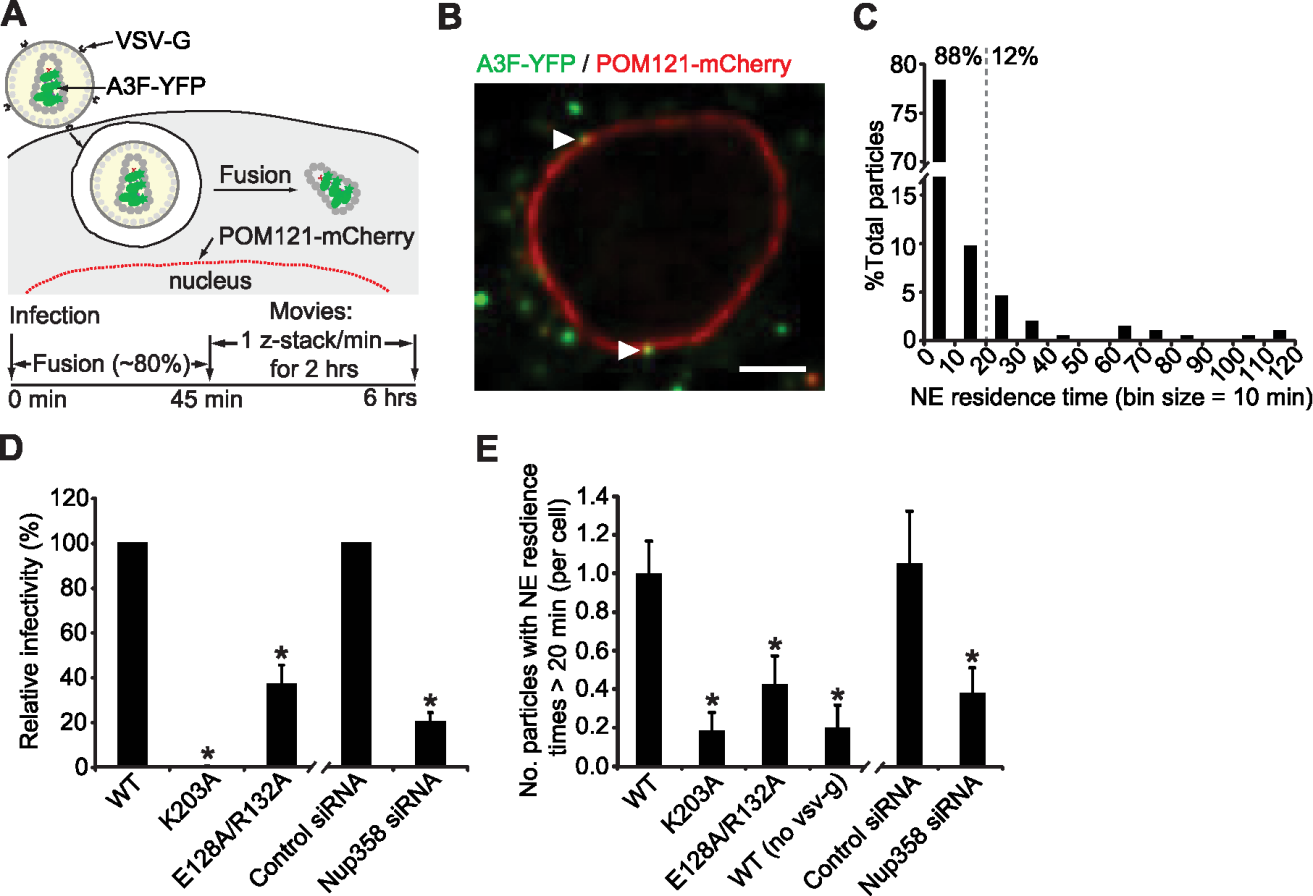
Kinetics of HIV-1 complex-NE association. (**A**) Experimental protocol. HeLa cells stably expressing POM121-mCherry were infected with VSV-G pseudotyped HIV-1 labeled with A3F-YFP. A z-stack centered near the equatorial region of the nucleus was acquired every 1 min for 2 hrs; movies were initiated from 45 min to 6 hrs post-infection. **(B)** Examples of A3F-YFP complexes (white triangles) that co-localize with the POM121-mCherry signal. Scale bar, 5 μm. **(C)** NE residence time analysis of A3F-YFP labeled HIV-1 complexes. The residence time for each HIV-1 complex that contacted the NE for at least 1 min (two consecutive frames) was determined and is plotted using 10 min bins. The percentage of these particles with NE residence times ≤ 20 min and > 20 min is shown; 194 particles from 23 cells were analyzed. **(D)** Relative infectivity of an HIV-1 GFP-reporter virus containing wild-type CA (WT), K203A, or E128A/R132A CA mutations (WT set to 100%; left). The relative infectivity of WT virus when the target cells are treated with control or Nup358 siRNA (control siRNA set to 100%; right) is shown. The percentage of GFP^+^ cells was determined by flow cytometry 48 hrs after infection. Error bars indicate the SD of 3–5 experiments. **(E)** The number of HIV-1 complexes that stably associate with the NE for > 20 min in the 2-hr long movies (per cell) is shown for WT, K203A, E128A/R132A, WT (no VSV-G) and for WT virus in cells treated with control siRNA or Nup358 siRNA. Error bars indicate standard error of the mean (SEM) of 19–27 cells. *, *P* ≤ 0.05, *t*-test.

The detailed kinetics of association of individual HIV-1 complexes with the NE and their residence time at the NE have not been examined, although association of a few viral complexes with the NE has been observed [2,36]. To address these questions, we generated HeLa cells that stably expressed POM121-mCherry, a nuclear pore marker (Fig 2A and 2B). We infected these cells with VSV-G pseudotyped virions containing A3F-YFP at a multiplicity of infection (MOI) of ∼1.1, defined as the number of proviruses per cell that expressed a GFP reporter gene (S1 Table). We previously estimated that in our imaging experiments 1 in 50 A3F-YFP labeled particles that entered each cell was imported into the nucleus [14]. To determine the fate of WT viral complexes that formed semi-stable (>1 min to < 20 min) and stable (>20 min) associations with the NE, we acquired time-lapse images (z-stack every 1 min for 2 hrs) and analyzed the residence time for each HIV-1 complex that associated with the NE for at least 1 min (2 consecutive frames; Fig 2C). Most WT HIV-1 complexes (171/194 = 88%, obtained from analysis of 23 cells) resided at the NE ≤ 20 min (avg. residence time = 5.0 min), whereas few complexes (23/194 = 12%) resided at the NE for >20 min (avg. residence time ≥ 51.4 min) (Fig 2C). Of these 23 particles, 10 particles also dissociated from the NE and no nuclear import was observed before the end of the observation time (120 min), suggesting that association with the NE for >20 min does not necessarily result in the nuclear import of the viral complex. These data strongly indicate that forming semi-stable associations with the NE (>1 min to < 20 min) is necessary but not sufficient for making stable associations lasting > 20 min.

To determine the role of HIV-1 CA in forming stable associations with the NE, we analyzed the association of HIV-1 complexes of a CA mutant (K203A) which forms unstable viral cores, and a CA mutant (E128A/R132A) that forms hyperstable viral cores [14,47]. We previously observed that these mutants were defective in nuclear import and in associating with the NE in a fixed-cell assay [14]. We previously proposed that an interaction between CA and the nuclear pore complex is essential for nuclear import, and that the hypostable mutant is defective in nuclear import because there is little or no CA associated with the viral complexes; in addition, the hyperstable mutant was defective in inducing structural/conformational changes in the viral core that are required to increase access to determinants that associate with the NE. We confirmed that these mutants had much lower levels of infectivity compared to wild-type HIV-1 (Fig 2D). We compared the ability of WT virus and CA mutants K203A and E128A/R132A to form stable associations with the NE (>20 min). We analyzed 19–27 cells for WT and CA mutants and observed that for both CA mutants the proportion of viral complexes that had NE residence times of >20 min was reduced to 0.2–0.4 particles/cell compared to 1.0 particle/cell for wild-type virus (Fig 2E). These observations indicated that the CA mutants that are defective in nuclear import are also defective in forming stable associations with the NE.

To identify host factors important for stable association of HIV-1 complexes with the NE, we carried out RNAi-mediated knockdown of Nup358 (S1A Fig), a nuclear pore protein that has been implicated to be directly [34,36,37,48] or indirectly [49] important for nuclear import and HIV-1 infectivity. Depletion of Nup358 significantly reduced infectivity compared to the control siRNA (Fig 2D; S1A Fig), steady-state NE association in a fixed-cell assay (S1B Fig), and nuclear import of HIV-1 complexes (S1C Fig). We determined the effects of Nup358 knockdown on cell viability using the ATPlite luminescence assay ∼48 hrs after transfection of control and Nup358 siRNAs; we did not find detectable levels of cytotoxicity at the time of our live-cell experiments, which were performed ∼48 hrs after siRNA transfection (S1D Fig). Similar to the CA mutations, Nup358 knockdown also reduced the proportion of HIV-1 complexes that stably associated with the NE to ∼0.4 particles/cell compared to 1.0 particle/cell for the siRNA control (Fig 2E). These results showed that both HIV-1 CA and Nup358 play important roles in the formation of stable associations between HIV-1 complexes and the NE. This result is consistent with the reduction in the steady-state level of HIV-1 complexes that were at the NE in Nup358-depleted cells (S1B Fig), but is in contrast to a recent report that showed a perinuclear accumulation of HIV-1 complexes at the NE in Nup358-depleted cells [36]. We also examined whether endosomal viral complexes can be transported to the NE and the extent to which they colocalized with the NE by analyzing endosomal viral complexes produced in the absence of VSV-G (Fig 2E). We found that the endosomal viral complexes were inefficient at forming stable associations with the NE compared to post-fusion complexes (∼0.2 vs.1.0/cell), indicating that stable association with the NE is largely a property of post-fusion viral complexes. The results also indicated that endosomal viral complexes may be associated with the NE at a low efficiency; we expect that our fusion efficiency is 80%, and 20% of the A3F-YFP labeled complexes may be trapped in endosomes. Since the endosomal complexes associated with the NE for >20 min with a ∼5-fold lower efficiency (∼0.2 vs. 1.0/cell), we estimate that only ∼4% of the viral complexes observed at the NE may be unfused viral complexes in endosomes.

To gain insights into transient associations of viral complexes with the NE that result in semi-stable associations (> 1 min), and to determine whether CA mutations and Nup358 knockdown influence the transient associations, we captured 1-min movies (10 frames/sec) of a single focal plane near the equatorial region of the nucleus from 45–120 minutes post-infection (mpi), after most virus particles (∼80%) fused and entered the cytoplasm (S2A Fig). A 1-μm wide mask of the NE was created using the POM121-mCherry signal to facilitate automated image analysis (S2B Fig). Within the focal volume (∼0.8 μm in height), particles that moved in the x, y, and z dimension could be followed, provided they generated tracks of sufficient length for analysis (≥ 5 steps). Overlapping of the first and last frames from the 1-minute movie indicated that the POM121-mCherry signal from the NE did not significantly move during the 1-min movie (S2C Fig; S1 Movie). We employed single-particle tracking combined with a population-based approach to analyze the movement of A3F-YFP labeled viral particles near the NE. A computer simulation to determine the length of time a complex would associate with the NE by random chance showed that very few (0.9%) of the randomly moving particles remained in contact with the NE for >5 sec (S2D Fig). We therefore defined contacts with the NE for >5 sec and < 1 min as transient associations. Qualitatively, the movies indicated that few HIV-1 complexes formed transient associations with the NE (S2E Fig [left and middle panels], S2 and S3 Movies), and most contacts of viral complexes with the NE lasted for <5 seconds (S2E Fig [right panel], S4 Movie).

To quantify transient NE association, we analyzed the association of HIV-1 complexes of wild-type HIV-1 and the CA mutants K203A and E128A/R132A in ∼20 cells per condition, and performed 3–5 independent experiments, constituting analysis of viral complexes from 60–100 cells for each experimental condition. We determined the longest consecutive residence time at the NE for each HIV-1 complex that colocalized with the POM121 mask for at least one frame (0.1 sec) during the observation time. We found that most of the 2,017 HIV-1 complexes that contacted the NE (92%) made contacts with the NE that remained for <5 sec, and only 8% formed transient associations (S2F and S2G Fig). Both CA mutants were defective for transiently associating with the NE; only 2.4 and 3.4% of the K203A and E128A/R132A complexes that contacted the NE, respectively, formed transient associations (S2F and S2G Fig). We never observed nuclear import of viral complexes in the movies used to analyze transient associations of viral complexes with the NE. Similar to the CA mutations, Nup358 depletion also reduced the proportion of HIV-1 complexes that transiently associated with the NE compared to the control siRNA (∼3% vs. ∼8%; S2F and S2G Fig). These results, in addition to the results described in Fig 2, indicate that both HIV-1 CA and Nup358 play important roles in the formation of both transient and stable associations between HIV-1 complexes and the NE.

### Kinetics of nuclear import and intranuclear movements

**Fig 3:**
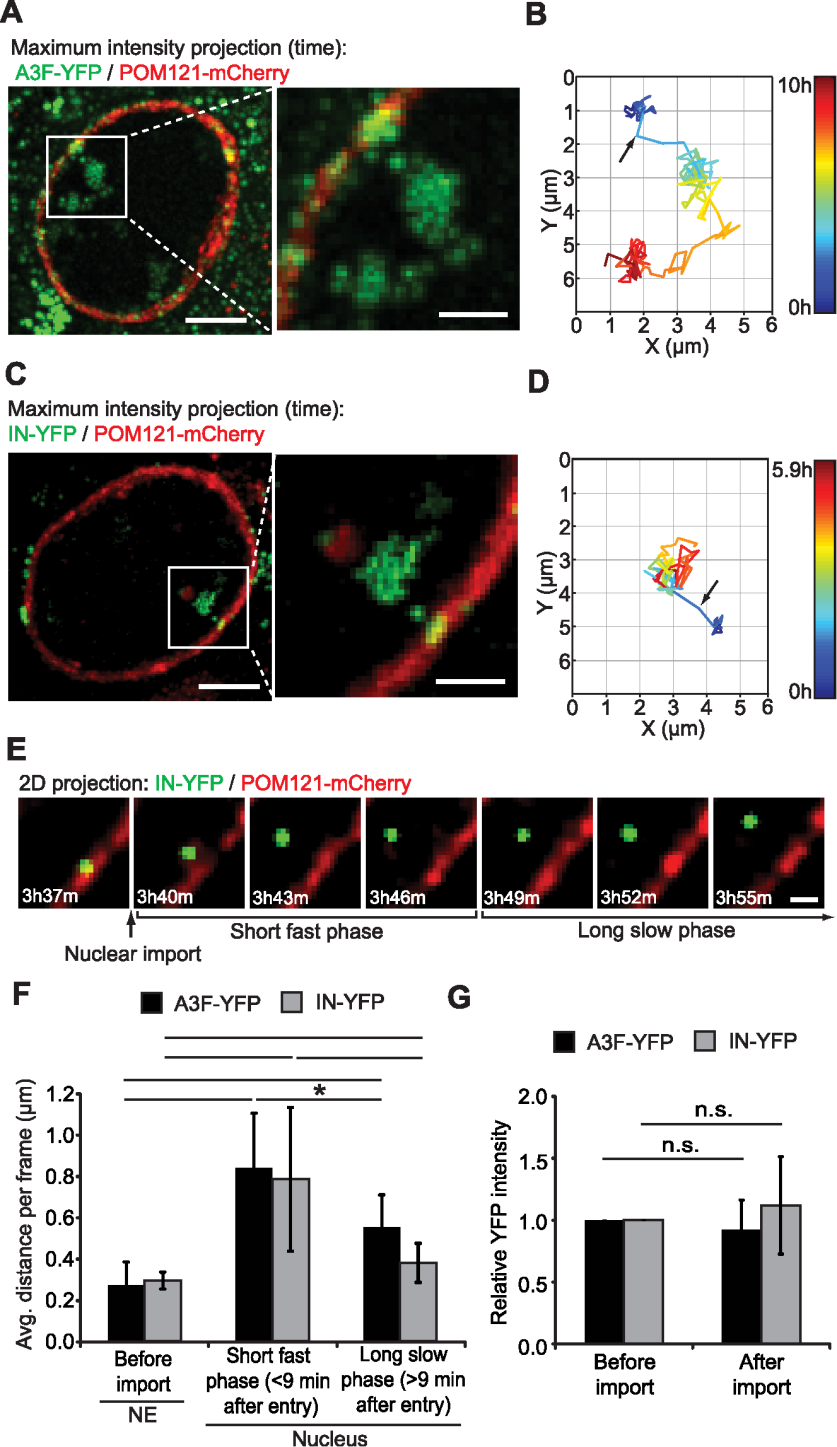
Translocation of HIV-1 complexes from the cytoplasm to the nucleus. (**A**) An A3F-YFP-labeled HIV-1 complex that was stably-associated with the NE (labeled by POM121-mCherry) for ∼3 hours prior to nuclear entry (S7 Movie). The z-slice in which the particle was located (as determined by 3D tracking) was extracted for each time point and then a maximum intensity projection of frames of the YFP and mCherry channels from the entire 10 hr time-lapse movie is shown. Scale bars, 5 μm (left) and 2 μm (right). **(B)** A 2D projection of the 3D track for the particle described in **(A)**. **(C)** An IN-YFP-labeled HIV-1 complex that was stably-associated with the NE for ∼1.5 hours prior to nuclear entry (S8 Movie) and **(D)** 2D projection of the 3D track for the particle described in **(C)**. For **(B)** and **(D)**, arrow indicates the first frame in which the HIV-1 complex was observed in the nucleus. **(E)** Composite images of the YFP and mCherry channels for the frame before and 6 frames after nuclear import for the IN-YFP labeled HIV-1 complex described in **(C)**. Scale bar, 1 μm. (**F**) The mobility of HIV-1 complexes before and after nuclear import. The average distance (μm) the particle moved per 3-min frame was determined for each A3F-YFP or IN-YFP complex prior to import, the 3 frames immediately following import (<9 min after particle was first detected in nucleus), and all subsequent frames. Error bars indicate SD of 7 import events for A3F-YFP complexes and 14 import events for IN-YFP complexes. **(G)** A3F-YFP and IN-YFP remain stably associated with viral complexes through nuclear import. The relative YFP intensities before (average YFP intensities for the 5 frames before import) and after nuclear import (average YFP intensities for the 5 frames after import) for each particle is shown; the YFP intensities before import were set to 1. *, *P* ≤ 0.05; **, *P* ≤ 0.01; n.s., not significant (*P* > 0.05), *t*-test.

Although nuclear HIV-1 complexes have been observed by fluorescence microscopy in living cells [2], the translocation of HIV-1 complexes from the cytoplasm to the nucleus has not been observed. To visualize the entry of HIV-1 complexes into the nucleus and gain insights into the kinetics of nuclear import and nuclear movements, we acquired z-stacks of cells every 3 min starting as early as 10 min after infection for up to 10 hrs. Each z-stack, which covered an area ∼4 μm in height, was centered near the equatorial plane of the nucleus; the top and bottom of the nuclei were not imaged to minimize photobleaching during the long movies. The movement of the nucleus during these long movies was corrected (S5 Movie) prior to 3D single-particle tracking of viral complexes. We observed 7 A3F-YFP labeled HIV-1 complexes enter the nucleus in ∼210 hrs of movies of 28 cells (Fig 3A and 3B and S3A and S3B Fig; Table 1 and S2 Table). Viral complexes were stably associated with the NE for ∼20 min (S6 Movie) to >3 hours (S7 Movie) immediately prior to import, indicating that the viral complexes that were observed to enter the nucleus had long residence times at the NE with wide variation in the length of time at the NE prior to import. In addition to the 7 particles that were observed to translocate from the cytoplasm to the nucleus, we observed an additional 37 A3F-YFP labeled complexes in the nuclei of 28 cells that were analyzed (∼1.6 HIV-1 complex/nucleus). Thus, translocation into the nucleus was visualized for ∼15% of the particles that were imported into the nuclei during the observation time (10-min to 10-hrs post-infection). After import, the viral complexes remained near the periphery of the nucleus throughout the observation time (Table 1 and S2 Table; S3C Fig), consistent with previous observations by us [14] and others [11,12,23]. The viral complexes exhibited long and variable residence times at the NE (Table 1 and S2 Table) and were imported 1–6 hrs post-infection, a time frame which is consistent with the kinetics of nuclear import [14,23].

**Table 1:** Summary of the dynamics of HIV-1 complexes at the NE and after nuclear import

Label	Nuclear penetration distance (μm)^1^		Distance from point of entry (μm)^1^		Time in cytoplasm (hours)^2^^,^^3^	NE residence time (hours)^2^	Observation time in nucleus (hours)^1^^,^^4^	Time of nuclear import (hpi)^2^^,^^5^
	Avg.	Max.	Avg.	Max.				
A3F-YFP or IN-YFP	1.4±0.4	2.1±0.6	2.3±0.8	3.2±1.1	2.8±1.9	1.5±1.6	3.8±2.7	4.3±2.6

^1^Values for the nuclear penetration distance, distance from point of entry, and observation time in nucleus are the average ± SD of 21 nuclear import events (S2 Table).

^2^Values for the time in cytoplasm, NE residence time, and time of nuclear import are the average ± SD of 26 nuclear import events (S2 Table).

^3^The time in cytoplasm represents the length of time between the time of infection and the time each viral complex arrived at the NE (see Fig 6A).

^4^ Observation time in nucleus represents the time a viral complex enters the nucleus until the end of the movie or until the viral complex exits from the z-stack.

^5^ hpi, hours post-infection.

Because A3F-YFP, a protein which can inhibit viral integration [20,21], could potentially alter the behavior of nuclear HIV-1 complexes, we also sought to observe the nuclear import of HIV-1 complexes using IN-YFP. We observed that viruses labeled with IN-YFP did not exhibit a defect in infectivity (S4A Fig); however, in our experiments, 8% of the viruses were labeled with IN-YFP, as determined by single virion microscopy analysis, and we cannot exclude the possibility that IN-YFP labeled virions are replication defective [23]. In this regard, previous studies have reported a modest decrease (∼2–3 fold) in infectivity of viruses labeled with Vpr-IN-GFP [24]. Although a lower proportion of virions were labeled with IN-YFP than with A3F-YFP (8% vs. 54%, respectively; S4B and S4C Fig), the average YFP fluorescence intensities of the labeled particles were similar (S4D Fig), suggesting the A3F-YFP and IN-YFP complexes would be detected with similar efficiencies. Upon infection of cells with equivalent amounts of A3F-YFP- or IN-YFP-labeled virions, we observed a similar number of YFP particles/cell, a similar percentage of cytoplasmic YFP particles that were at the NE, and a similar percentage of YFP particles that were in the nucleus throughout the time-course (S4E–S4G Fig; *P* > 0.05, *t*-test). Thus, ∼7-fold higher amount of virus was required to achieve a similar number of YFP-labeled particles in infected cells using IN-YFP compared to A3F-YFP ([100/8] ÷ [100/54] = 6.75), but IN-YFP and A3F-YFP remained associated with HIV-1 complexes in infected cells with a similar efficiency.

Next, we visualized the translocation of 14 IN-YFP labeled HIV-1 complexes from the NE to the nucleus in 910 hrs of movies of 91 cells (Table 1 and S2 Table; Fig 3C and 3D; S8 Movie). After import, both A3F-YFP and IN-YFP labeled viral complexes remained near the periphery of the nucleus throughout the observation time (average and maximum nuclear penetration distances are 1.4 ± 0.4 μm and 2.1 ± 0.6 μm from NE, respectively [Table 1 and S2 Table]), but moved away from the point of nuclear entry (average and maximum distances of 2.3 ± 0.8 and 3.2 ± 1.1 μm, respectively). The A3F-YFP and IN-YFP complexes exhibited long and variable NE residence times prior to nuclear import (average 1.5 ± 1.6 hours), and were imported an average of 4.3 ± 2.6 hours after infection. Importantly, no significant differences were observed between A3F-YFP and IN-YFP labeled viral complexes regarding nuclear penetration distance, distance from point of entry, time in cytoplasm prior to NE association, NE residence time, and time of nuclear import (*P* > 0.05, *t*-test or Mann Whitney test; S2 Table), indicating that the method of labeling viral complexes did not have any discernible effect on these aspects of viral replication. Immediately following nuclear import, there was a brief fast phase (<9 min [3 frames]) in which the viral complexes exhibited higher mobility, followed by a second phase of slower mobility for the remainder of the observation time (Fig 3E and 3F). On average, the YFP intensity was similar just before and after nuclear import, indicating that both A3F-YFP and IN-YFP remained stably associated with the viral complexes through nuclear import (Fig 3G).

### Long NE residence time is a requirement for nuclear import

We analyzed nuclear viral complexes for which the translocation from the NE to the nucleus was not observed. These particles may have entered the nucleus from above or below the z-stack of observed nuclear volume; alternatively, these particles may have entered the nucleus with a very short NE residence time (< 3 min) such that their NE docking was not captured in the 3-min/frame movies. We identified 80 such particles in movies discussed in Fig 3 as well as in movies that were manually analyzed to determine NE residence time (discussed below). Particles that were in the nucleus at the start of the movies were excluded from this analysis. We found that 22 of these nuclear viral complexes first appeared in the bottom z-slice, 57 first appeared in the top z-slice, and 1 appeared in the center of the z-stack but could not be confidently tracked because of low signal intensity. Viral complexes that entered the nucleus with very short NE residence times (< 3 min), would be expected to appear in the equatorial plane of the nucleus and it would not be possible to track them to the NE or the top or bottom z-slices. With the exception of one dim particle, 79 of these 80 particles in the nucleus were tracked to the Z-slice above or below the equatorial Z-stack. Thus, we conclude that most (if not all) nuclear viral complexes for which the nuclear entry was not observed (S3A Fig) entered the nucleus from above or below the z-stack. In addition, we observed 30 viral complexes that exhibited long NE residence times and entered the nucleus (21 tracked particles described above and 9 additional particles from experiments discussed below; S2 Table). Therefore, <1/110 nuclear viral complexes may have entered the nucleus with short NE residence times (<3 min), strongly supporting the view that a long NE residence time is a requirement for successful nuclear import.

### Nuclear movement of HIV-1 complexes

**Fig 4:**
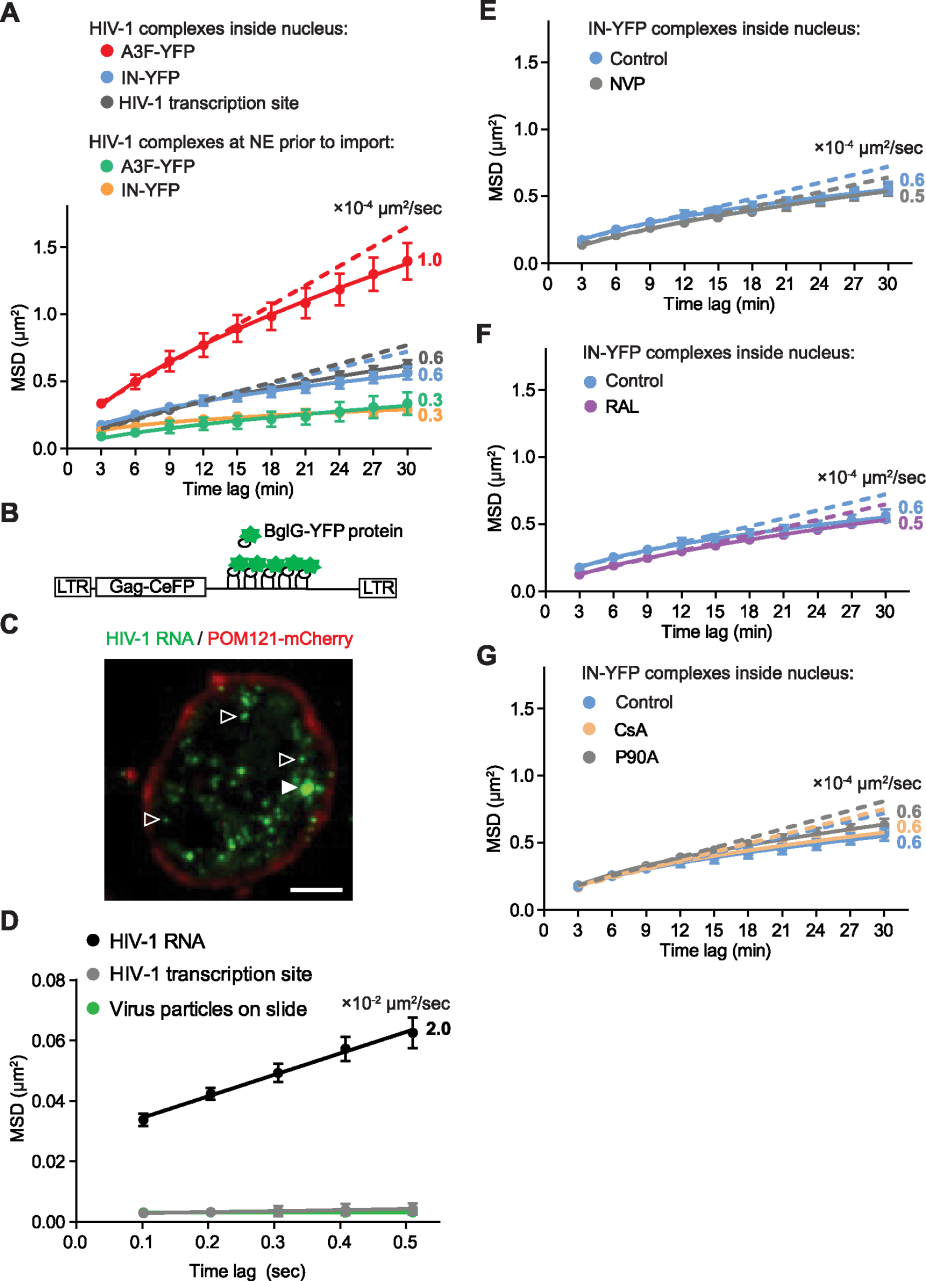
Analysis of intranuclear movements of A3F-YFP and IN-YFP labeled viral complexes and HIV-1 transcription sites. **(A)** Ensemble mean square displacement (MSD) analysis of the movement of A3F-YFP and IN-YFP labeled HIV-1 complexes at the NE prior to import and inside the nucleus, as well as their comparison to the movement of HIV-1 transcription sites (described below). Dotted lines are straight line fits using the first four time lags and extrapolated to the last time lag. The curves (solid lines) represent power law fits to the MSD values. The diffusion coefficients (× 10^−4^ μm^2^/sec) were calculated using the MSD values and are shown next to each curve. **(B-D)** Detection of HIV-1 transcription sites in infected cells. **(B)** A schematic illustrating an HIV-1 genome containing 18 RNA-binding stem-loops that can be specifically labeled by the BglG-YFP fusion protein. **(C)** A deconvolved image of a POM121-mCherry-expressing HeLa cell infected with HIV-1 containing the engineered viral genome described in **(B),** transfected 5 days later with the BglG-YFP fusion protein (HIV-1 RNA, green; POM121-mCherry, red), and imaged the next day; solid triangle indicates HIV-1 transcription site; empty triangles indicate HIV-1 RNA. Scale bar, 5 μm. **(D**) Ensemble MSD analysis of the movement of HIV-1 RNA, HIV-1 transcription sites, and virus particles on a slide. Time-lapse images of infected cells expressing HIV-1 RNA or virus particles on a slide were acquired at 10 frames/second for 1-min. Single particle tracking of ∼90 HIV-1 RNA particles from 3 cells (totaling 37,437 steps), 5 HIV-1 transcription sites from 5 cells (totaling 2,995 steps), 113 HIV-1 particles on a slide (totaling 46,202 steps) was performed followed by MSD analysis. Lines indicate straight line fits to the MSD values. The diffusion coefficient (× 10^−2^ μm^2^/sec) was calculated using the MSD values and is shown next to the HIV-1 RNA curve; movement of HIV-1 transcription sites and virus particles on slide were not detected at these time lags. **(E-G)** Ensemble MSD analysis of the movement of IN-YFP labeled nuclear HIV-1 complexes after treatment of target cells with NVP **(E)**, RAL **(F)**, or CsA and CypA-binding CA mutant P90A **(G)**. HeLa cells stably expressing POM121-mCherry were infected with IN-YFP labeled virus and ∼10 hr-long movies (1 frame/3 min) of cells with 1 or more nuclear viral complexes initiated ∼2 hrs after infection were acquired. The control values in **(E-G)** are replotted from the IN-YFP labeled HIV-1 complexes inside the nucleus described in **(A)**. IN-YFP labeled nuclear complexes (≥ 10 complexes resulting in a total of >30 hrs of movement for each condition) were analyzed. The diffusion coefficients (× 10^−4^ μm^2^/sec) were calculated using the MSD values and are shown next to each curve. Error bars represent 95% confidence intervals.

To gain further insight into the movement of viral complexes in the long slow phase, we performed an ensemble mean square displacement (MSD) analysis of nuclear viral complexes (Fig 4A). The diffusion rates of nuclear A3F-YFP labeled and IN-YFP labeled viral complexes were low (1 and 0.6 × 10^−4^ μm^2^/sec, respectively), but were 2- to 3-fold higher than when they were docked at the NE prior to import (0.3 × 10^−4^ μm^2^/sec). A linear relationship between MSD values and time indicate free diffusion. However, the MSD graph lines indicate a sub-linear relationship between the MSD values and time, indicating that the viral complexes inside the nucleus exhibited restricted diffusion. The diffusion coefficients of A3F-YFP and IN-YFP labeled HIV-1 complexes in the long slow phase were within twofold of the diffusion coefficients that have been reported for genes (reviewed in [50]), supporting the hypothesis that the viral complexes are tethered to chromatin. Interestingly, the diffusion rate of A3F-YFP labeled viral complexes was twofold higher than that of IN-YFP labeled viral complexes. The observation suggests that virion incorporation of A3F-YFP alters the structure of the viral complex in a way that reduces its ability to tether to the host chromatin. This effect of A3F may be related to the ability to A3F to inhibit HIV-1 integration by interfering with the IN-mediated 3’ processing reaction [20,21].

To further examine the movement of nuclear viral complexes in the long slow phase, we sought to determine whether their movement would be similar to nascent HIV-1 RNA that is tethered to the proviral DNA transcription sites in chromatin until transcription is completed and the RNA is released. If the viral complexes exhibited a diffusion rate that is faster than that of proviral transcription sites, the result would suggest that the viral complexes dissociate/reassociate with multiple chromatin sites before integration and provirus formation. We detected HIV-1 RNA transcribed from the proviral DNAs using a previously described strategy [51]. Briefly, the viral genome was engineered to encode 18 RNA stem-loops that are specifically recognized by the *Escherichia coli* BglG protein that was tagged with YFP (Fig 4B). It has been previously shown that the strongest RNA signals in the nuclei represent nascent RNA transcripts that are retained at the transcription site until they are released [52–54]. Single cell clones containing one or two proviruses encoding stem-loops that bind to BglG were selected and expanded, the integrated proviral transcription sites were identified by detection of the brightest RNA signals in the nuclei after expression of the BglG-YFP fusion protein (Fig 4C). The movements of 11 transcription sites in living cells (totaling 47 hours of movement) were analyzed. The diffusion coefficient of the HIV-1 transcription sites (0.6 × 10^−4^ μm^2^/sec; Fig 4A) was nearly identical to that of IN-YFP labeled viral complexes and within 2-fold of the A3F-YFP labeled viral complexes, and in agreement with previously reported diffusion coefficients of genes (reviewed in [50]). The results support the hypothesis that the viral complexes are tethered to chromatin and that the movement in the long slow phase was largely due to the movement of the chromatin.

We also observed several faint RNA spots in the cells that contained HIV-1 proviruses and expressed the BglG protein, which we hypothesize are HIV-1 ribonucleoprotein complexes (Fig 4C; [51,55]). These RNA spots exhibited much faster movement than the RNA transcription sites, and their movements could not be analyzed from the 1 frame/3 min movies. We captured additional movies at 10 frames/sec, performed single particle tracking followed by MSD analysis of their movements (Fig 4D). The results indicated a diffusion rate of 2 × 10^−2^ μm/sec, which is significantly faster than the diffusion rate of HIV-1 transcription sites (0.6 × 10^−4^ μm/sec; Fig 4A); this diffusion rate is in general agreement with previously reported diffusion coefficients for nuclear ribonucleoprotein complexes [54,56]. Because of the significantly slower movements of HIV-1 transcription sites, their MSD plot was not significantly different from immobile virus particles on a glass slide at these time lags. Importantly, the MSD analysis can clearly distinguish between HIV RNA transcription sites and HIV ribonucleoprotein complexes.

Next, we compared the intranuclear movements of viral complexes in the long slow phase in cells that were treated with RT inhibitor NVP (Fig 4E), IN inhibitor RAL (Fig 4F), or CsA, which disrupts CA-CypA interaction (Fig 4G). The results showed that these treatments had no effect on the intranuclear movements of viral complexes, suggesting that the absence of reverse transcription does not affect the apparent tethering of viral complexes with chromatin, and that unintegrated viral complexes exhibit movements that are similar to those of chromosomal HIV-1 RNA transcription sites. Finally, the intranuclear viral complexes in the presence of CsA presumably did not have any CypA associated with them; the presence or absence of CypA in association with the intranuclear viral complexes did not have any impact on their nuclear movements. Similarly, the CA mutant P90A, which is defective for binding to CypA, exhibited a diffusion rate that is similar to the WT viral complexes and viral complexes in CsA-treated cells (Fig 4G).

### Nuclear movement and nuclear location of HIV-1 complexes is not dependent on LEDGF/p75

**Fig 5:**
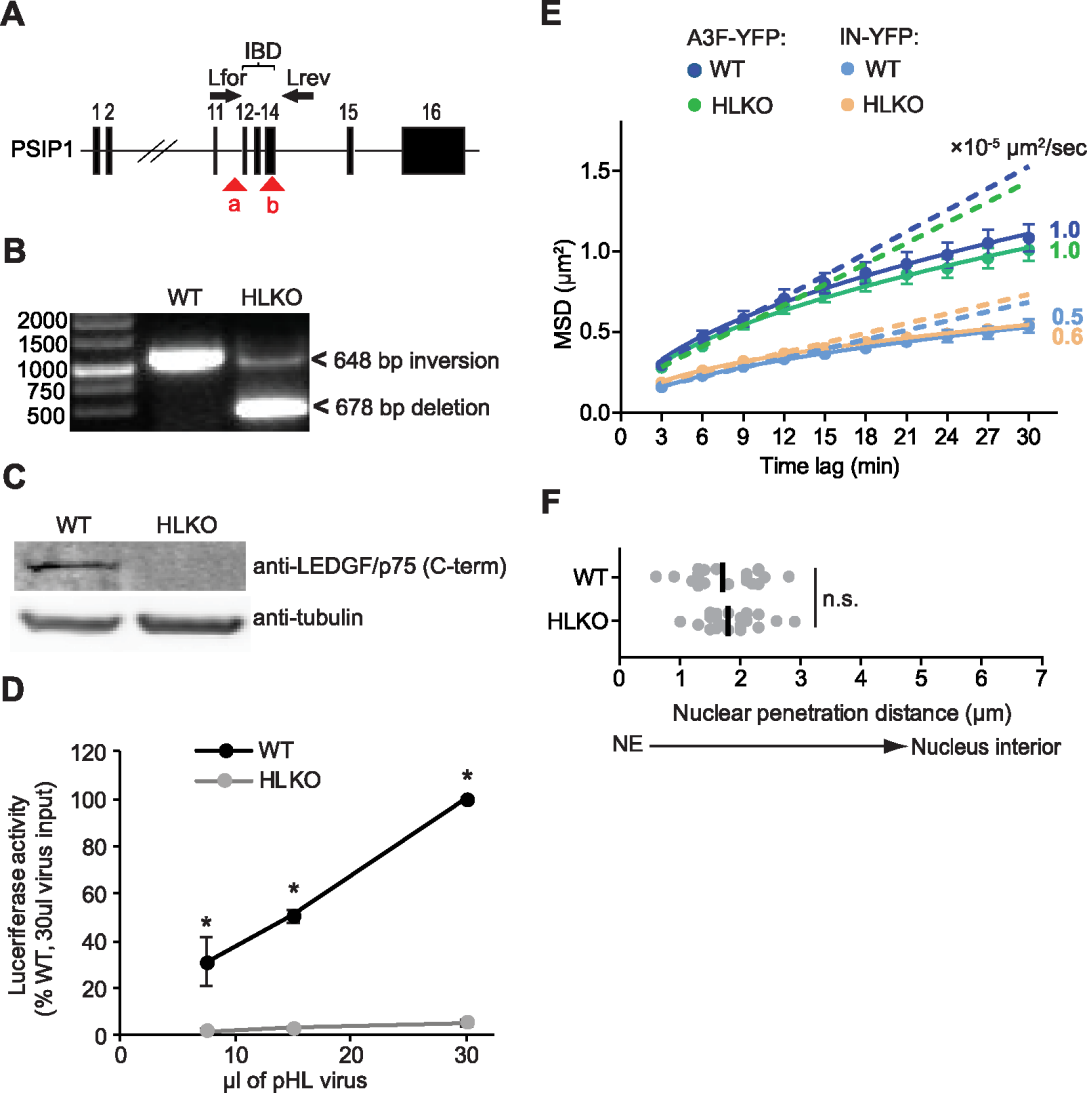
Analysis of intranuclear movements of A3F-YFP and IN-YFP labeled viral complexes in the presence or absence of LEDGF/p75. **(A-D)** Generation of a LEDGF/p75 knockout HeLa cell line (HLKO) in which the integrase binding domain (IBD) of LEDGF/p75 was disrupted using CRISPR/cas9. **(A)** PSIP1 locus on chromosome 9, which codes for LEDGF/p75, is shown with exons (black boxes) labeled 1 to 16. Locations of the gRNA binding sites are shown as red triangles (a and b). Primers binding to the outside of the integrase binding domain (IBD), used for PCR amplification and identification of single cell clones, are labeled Lfor and Lrev. **(B)** PCR analysis of LEDGF/p75 knock out clone HLKO versus wild-type HeLa cells (WT). Expected band sizes are 1158 bp for undeleted IBD, versus ∼480 bp for deleted IBD. **(C)** WT or HLKO cell lysates were analyzed by western blot using antibodies that detect the C-terminal region of LEDGF/p75 or α-tubulin. **(D)** Relative infectivity of an HIV-1 luciferase-reporter virus (pHL) in WT or HLKO cells. The luciferase activity was determined 48 hrs after infection; luciferase activity in WT cells infected with 30 μl pHL virus was set to 100%. *, *P* ≤ 0.05; *t*-test. **(E)** Ensemble MSD analysis of the movements of intranuclear A3F-YFP and IN-YFP labeled HIV-1 complexes in WT and HLKO cells. WT or HLKO cells expressing POM121-mCherry were infected with A3F-YFP or IN-YFP labeled virus and ∼10 hr-long movies (1 frame/3 min) of cells with 1 or more nuclear viral complexes initiated ∼2 hrs after infection were acquired. Dotted lines are straight line fits using the first four time lags and extrapolated to the last time lag. The curves (solid lines) represent power law fits to the MSD values. The diffusion coefficients (× 10^−4^ μm^2^/sec) were calculated using the MSD values and are shown next to each curve; error bars represent 95% confidence intervals. **(F)** Average nuclear penetration distance for each viral complex in WT (n = 20) and HLKO cells (n = 20) described in **(E)**; the nuclear penetration distances for A3F-YFP and IN-YFP labeled viral complexes were not different (*P* > 0.05; Mann-Whitney test) and were combined for these experiments; black lines indicate median nuclear penetration distance; n.s., not significant (*P* > 0.05; Mann-Whitney test).

Lens epithelium-derived growth factor/p75 (LEDGF/p75) is a nuclear protein that binds to nucleosomes of transcriptionally active genes and is thought to tether proteins or protein complexes to chromatin (reviewed in [57,58]). LEDGF/p75 binds tightly to HIV-1 IN and facilitates HIV-1 replication by directing integration into transcriptionally active genes [59]. We sought to determine whether interaction between LEDGF/p75 and HIV-1 IN is essential for tethering of viral complexes to chromatin during the long slow phase of nuclear movement. We generated HeLa cells in which a portion of LEDGF/p75 encoding gene (PSIP1) that codes for the integrase binding domain of LEDGF/p75 (IBD) was deleted by using CRISPR/cas9 (Fig 5A). Transduction of HeLa cells with CRISPR/cas9 and two different gRNAs resulted in a HeLa cell clone (HLKO) with deletion of a 678-bp fragment in two alleles, and inversion of a 648-bp fragment in another allele, resulting in a complete knockout of the IBD (Fig 5B and S5 Fig). Western blotting analysis with an antibody targeting an epitope near the C-terminus of LEDGF/p75 confirmed that LEDGF/p75 expression was undetectable (Fig 5C). Infection of the HLKO cells with a virus that expresses a luciferase reporter gene showed that luciferase expression was reduced by ∼20-fold (Fig 5D). We infected the HLKO cells with A3F-YFP and IN-YFP labeled virions, and compared the movements of nuclear viral complexes to those in WT HeLa cells (Fig 5E). The results showed that deletion of LEDGF/p75 from the infected cells did not have any effect on the movement of A3F-YFP or IN-YFP labeled nuclear viral complexes in the long slow phase. These results suggest that LEGDF/p75 is not required for the proposed tethering of viral complexes to chromatin and that other host factors may be involved in this interaction with the viral complexes. Finally, we compared the nuclear penetration distance of viral complexes in WT and HLKO cells (Fig 4F). We found that the viral complexes were located near the periphery of the nuclei, regardless of the presence or absence of LEDGF/p75 (median penetration distances of 1.7 and 1.8 μm, respectively). Overall the results indicated that the interaction of viral complexes with LEDGF/p75 is not a determinant of the proposed tethering of viral complexes to chromatin or their nuclear penetration distance.

### Regulation of the dynamics of NE docking and nuclear import by CypA

**Fig 6:**
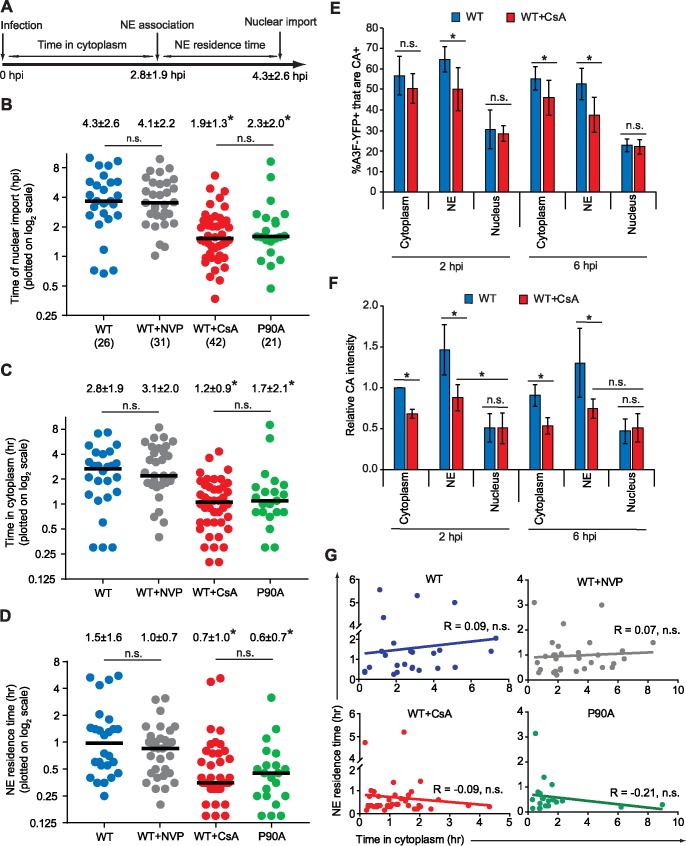
CA-CypA interactions, but not reverse transcription, regulate nuclear import. (**A**) A schematic illustrating that the time of nuclear import consists of the time in the cytoplasm prior NE docking (time between infection and NE association) and NE residence time prior to nuclear import (time between NE association and nuclear import). The average time of NE association and nuclear import for the WT complexes (described below) are shown here for reference. **(B-D)** The time of nuclear import **(B)**, time in cytoplasm **(C)**, and NE residence time **(D)** for each viral complex that entered the nucleus is shown. For these experiments, the nuclear import of WT complexes in untreated cells, WT complexes in the presence of NVP, WT complexes in the presence of CsA, and P90A CA mutant complexes were detected manually from analysis of 10-hr long movies initiated 10 min after infection (1 frame/3 min). Nuclear import events for WT complexes in untreated cells also includes tracked viral complexes (S2 Table). Numbers below sample name in **(B)** indicate the number of nuclear import events analyzed. For **(B-D)**, the average values ± SD are shown above each sample; black lines indicate median values; *, significantly different than WT and WT+NVP (*P* ≤ 0.05; Mann-Whitney test); n.s, not significant (*P* > 0.05; Mann-Whitney test). **(E-F)** Characterization of WT viral complexes labeled with A3F-YFP in infected cells treated without (WT) or cells treated with CsA (WT+CsA). HeLa cells treated with and without CsA were infected with VSV-G pseudotyped HIV-1 labeled with A3F-YFP and fixed 2 or 6 hrs post-infection (hpi). **(E)** The percentage of A3F-YFP^+^ signals that are CA^+^ and **(F)** relative CA signal intensity (average CA signal intensities associated with A3F-YFP signals in the cytoplasm at 2 hpi was set to 1). Error bars indicate the SD of 4 experiments; an average of ∼1400 particles from ∼100 cells were analyzed for each condition. *, *P* ≤ 0.05; n.s., not significant (*P* > 0.05; *t*-test). **(G)** Scatter plots of the NE residence times (hrs) and time in cytoplasm (hrs) for each viral complex described in **(B-C)**. Linear trend lines for each population was determined (solid lines); Pearson correlation values are shown for each population; n.s., indicates no significant correlation between NE residence time and time in cytoplasm (*P* > 0.05).

We sought to determine whether reverse transcription and/or CypA associated with viral complexes regulate the dynamics of nuclear import. We previously showed that inhibiting reverse transcription with reverse transcriptase (RT) inhibitor nevirapine (NVP) or using a catalytic site mutant of RT did not influence the efficiency of nuclear import, leading us to conclude that reverse transcription was not required for nuclear import [14]. However, our previous studies did not rule out the possibility that reverse transcription can regulate the timing of NE docking and/or nuclear import. In addition, the CA-CypA interaction has been shown to modulate capsid uncoating and alter the dependence on some nuclear pore proteins for nuclear import [25,29,32–34]. Therefore, we sought to determine whether CypA binding and/or reverse transcription can regulate the timing of NE docking and nuclear import. For these studies, we identified 9 additional nuclear import events (6 labeled with IN YFP and 3 labeled with A3F-YFP) by manual analysis of new movies of infected cells (1 frame/3 min from 10 min– 10 hrs after infection) in addition to 17 of the 21 tracked viral complexes described above, providing a total of 26 viral particles that were observed to enter the nucleus in untreated cells (for this analysis 4 A3F-YFP labeled particles that were at the NE at the beginning of the movies were excluded [S2 Table]). Determining the time of nuclear import and docking of viral complexes at the NE relative to the time of infection allowed us to determine the length of time the viral complexes were in the cytoplasm (time in cytoplasm) and the length of time the viral complexes were associated with the NE prior to import (NE residence time) (Fig 6A). In addition to the 26 particles analyzed from untreated cells, we analyzed 31, 42, and 21 nuclear import events of viral complexes in cells treated with NVP to block reverse transcription, in cells treated with CsA to disrupt the CA-CypA interaction, and viral complexes derived from CypA-binding mutant P90A, respectively (Fig 6B). We observed that compared to untreated cells, NVP treatment did not significantly alter the time in cytoplasm, NE residence time, and time of import, indicating that inhibiting reverse transcription did not regulate nuclear import (Fig 6B–6D). However, when compared to untreated cells, disruption of CA-CypA interaction by treatment of target cells with CsA or the CypA-binding CA mutant P90A significantly decreased the time of import; the mean time of import for wild-type viral complexes was 4.3 ± 2.6 hours post-infection (hpi), which was reduced to 1.9 ± 1.3 hpi and 2.3 ± 2.0 hpi for viral complexes in CsA-treated cells or cells infected with the P90A mutant of CA, respectively (Fig 6B). These results indicate that CypA binding to viral capsid results in slowing down nuclear import, whereas disruption of the CA-CypA interaction results in faster nuclear import. The observation that CsA treatment and the P90A mutation had very similar effects on the time of import strongly indicates that the faster nuclear import is not the result of an indirect effect of CsA treatment or an indirect effect of the P90A mutation on the viral capsid structure/function.

Next, we determined if the faster nuclear import in CsA-treated cells was due to a decrease in the cytoplasmic residence time prior to NE association and/or a decrease in the NE residence time. We found that treatment with CsA led to a decrease in the time in cytoplasm (1.2 ± 0.9 hpi vs. 2.8 ± 1.9 hpi, *P* = 0.0001; Fig 6C) as well as a decrease in the NE residence time (0.7 ± 1.0 hpi vs. 1.5 ± 1.6 hpi, *P* = 0.0001; Fig 6D). Similarly, the P90A mutation also led to a decrease in the time in cytoplasm (1.7 ± 2.1; *P* = 0.0091) as well as a decrease in the NE residence time (0.6 ± 0.7 hpi; *P* = 0.0010). A comparison of the shorter times of import, time in cytoplasm, and NE residence time in CsA-treated cells and the P90A mutant indicated that there were no significant differences in these two conditions, further supporting a direct role for CA-CypA interaction in the regulation of nuclear import.

We also determined whether CsA treatment or the P90A mutation affected the efficiency of nuclear import in HeLa cells by determining the level of nuclear import in fixed cells at early (2 and 6 hpi) and late (24 hpi) time points. The level of nuclear import was higher at 2 and 6 hpi with CsA treatment or the P90A mutation, which is consistent with our results obtained from the visualization of nuclear import in living cells, but was similar at 24 hpi (S6A Fig). In addition, the infectivity of WT virus in untreated cells, WT virus in the presence of CsA treatment, or the P90A mutant virus was similar in HeLa cells (S6B Fig). These results indicate that although the speed of nuclear import increases with disruption of the CA-CypA interaction, the efficiency of nuclear import for viral complexes and infectivity is similar with or without disruption of the CA-CypA interaction.

Our results indicated that lower levels of CA are associated with nuclear viral complexes compared to NE-associated viral complexes (Fig 1F and 1G), suggesting that a portion of viral core uncoating occurs during nuclear import. Previous studies have suggested the CypA stabilizes viral complexes by slowing down uncoating, and that CsA treatment may accelerate viral core uncoating [60]. To explore the mechanism by which the CypA-CA interaction influences the kinetics of nuclear import, we determined the level of CA associated with viral complexes at 2 and 6 hours post-infection in the presence or absence of CsA by immunofluorescence staining (Fig 6E and 6F). At the 2-hour time point, the percentage of cytoplasmic and nuclear A3F-YFP signals that colocalized with CA was similar with and without CsA treatment (Fig 6E), and a modest reduction in the percentage of NE-associated A3F-YFP signals that colocalized with CA was observed with CsA treatment (65% vs. 50%; *P* < 0.05). At the 6-hour time point, the proportions of cytoplasmic and NE-associated A3F-YFP signals that colocalized with CA were reduced with CsA treatment. There were no differences in the proportion of nuclear A3F-YFP signals that colocalized with CA at 2 or 6 hours after infection. Next, we compared the CA signal intensities that colocalized with A3F-YFP signals in the cytoplasm, NE, and the nucleus with and without CsA treatment at the 2-hour and 6-hour time points (Fig 6F). We observed that the CA signal intensities that colocalized with the A3F-YFP signals were reduced in the cytoplasm and at the NE at both 2-hour and 6-hour time points, but there were no differences in CA signal intensities associated with the A3F-YFP signals in the nuclei. The reduced CA signal intensities in the cytoplasm and at the NE in CsA-treated cells indicate that CsA treatment facilitated faster viral core uncoating, which may have resulted in faster docking at the NE (reduced time in cytoplasm), as well as reduced NE residence time prior to nuclear import.

We sought to determine whether a longer time in cytoplasm is correlated with changes in the viral core that increase speed of nuclear import (reduce NE residence time). As we noted earlier, most viral complexes that formed semi-stable and stable associations with the NE dissociated from it and were not imported into the nucleus. It is not known whether the viral complexes that dissociated from the NE are defective and cannot reassociate with the NE, or whether successive cycles of NE association/dissociation contribute to the dynamics of nuclear import of viral complexes, and result in shorter NE residence times and faster nuclear import. If viral complexes that are imported undergo successive cycles of association/dissociation at the NE, a longer time in cytoplasm might be correlated with more cycles of NE association/dissociation; therefore, we determined whether the time in cytoplasm correlated with a shorter NE residence time prior to import. We found no correlation between the length of time in cytoplasm and NE residence time for each viral complex that we observed enter the nucleus within the four different populations of viral complexes (WT complexes, WT+CsA, P90A, and WT+NVP population; Fig 6G; Pearson Correlation, *P* > 0.05). The lack of correlation between time in cytoplasm and NE residence time suggests that viral complexes in the cytoplasm, or those that make multiple transient contacts with the NE without being imported, did not accumulate any structural/conformational changes that shortened the NE residence time. Overall, these results suggest that NE docking and translocation from the NE to the nucleus are independent events, both of which are regulated by the CA-CypA interaction.

**Fig 7:**
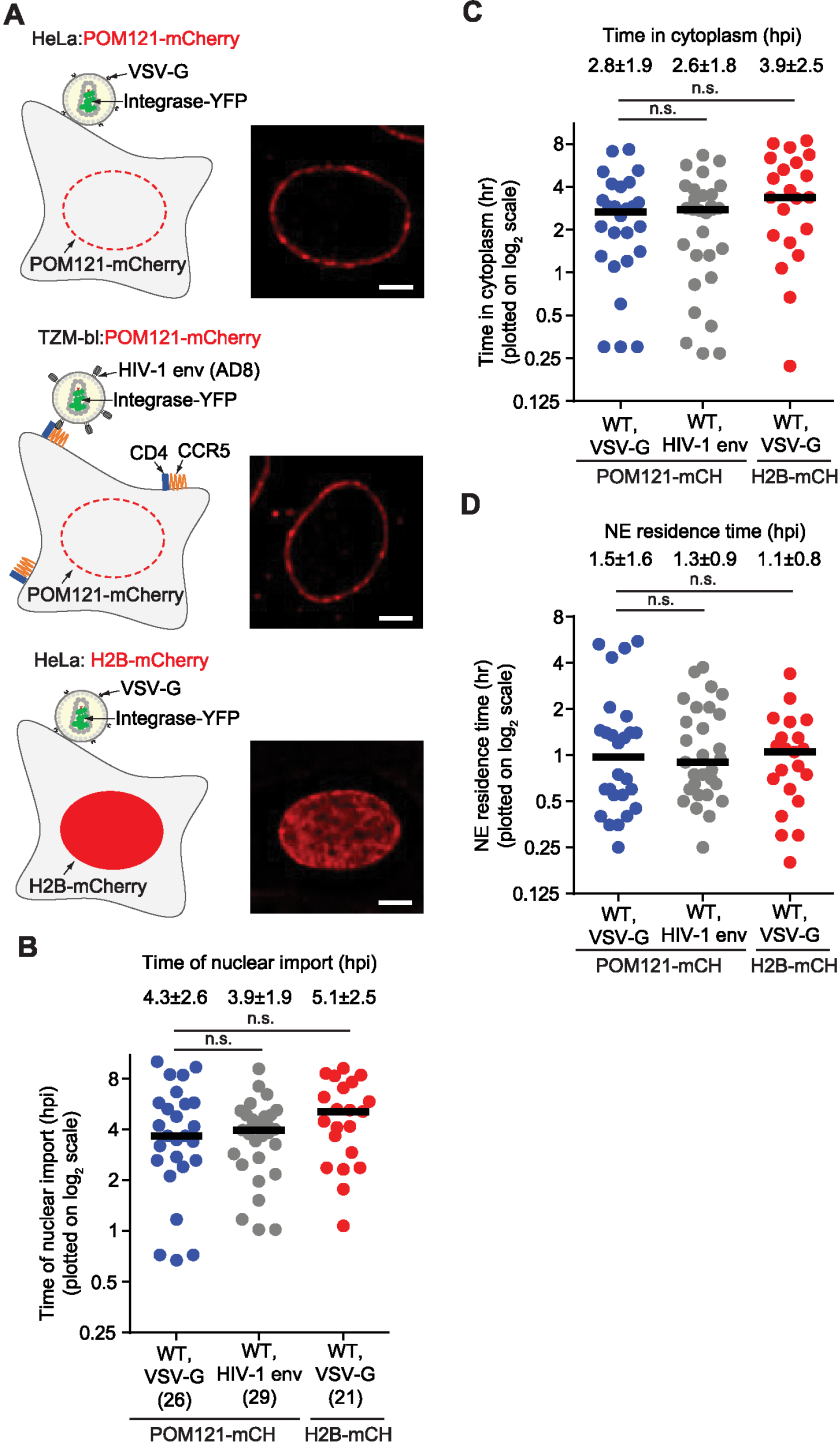
The kinetics of nuclear import of HIV-1 virions with VSV-G or HIV-1 envelope is similar and is not altered by expression of POM121-mCherry. **(A)** Schematic of a POM121-mCherry expressing HeLa cell infected with a VSV-G pseudotyped virion (top panel), a POM121-mCherry expressing TZM-bl cell, which express high levels of CD4 and CCR5, infected with a virion containing HIV-1 envelope (middle panel), or an H2B-mCherry expressing HeLa cell infected with a VSV-G pseudotyped virion (bottom panel). Example deconvolved images of cells expressing POM121-mCherry or H2B-mCherry are shown to the right of the schematics. Scale bar, 5 μm. **(B-D)** The time of nuclear import **(B)**, time in cytoplasm **(C)**, and NE residence time **(D)** for each viral complex that entered the nucleus is shown. Viral complexes were detected manually from analysis of 10-hr long movies initiated 10 min after infection (1 frame/3 min). The nuclear import events for the VSV-G pseudotyped virions infected in POM121-mCherry expressing cells are replotted from Fig 6 for comparison. Numbers below sample name in **(B)** indicate the number of nuclear import events analyzed. For **(B-D)**, the average values ± SD are shown above each sample; black lines indicate median values; n.s, not significant (*P* > 0.05; Mann-Whitney test).

It has been reported that infectivity of CA mutant N74D is independent of TNPO3 when using VSV-G-mediated entry, but dependent on TNPO3 when using HIV-1 envelope (Env) mediated entry [61]. To determine whether virions that use the HIV-1 Env and fuse at the plasma membrane have different kinetics of NE docking and nuclear import, we compared the kinetics of HIV-1 NE docking and nuclear import in HeLa cells for VSV-G mediated entry to HIV-1 Env mediated entry (Fig 7A, upper and middle panels). For these experiments, we constructed a TZM-bl HeLa cell line that expresses POM121-mCherry (Fig 7A middle panel); TZM-bl cells express HIV-1 Env receptor CD4 and co-receptor CCR5 on their cell surface and virions with HIV-1 Env can infect these cells through fusion at the plasma membrane. We did not find any significant difference between VSV-G pseudotyped virions and HIV-1 Env virions with regard to the time of nuclear import (Fig 7B), time in cytoplasm (Fig 7C), or NE residence time (Fig 7D), indicating that the envelope used for entry into cells does not have any significant effect on the kinetics of NE docking or nuclear import.

Finally, we sought to rule out the possibility that expression of POM121-mCherry, a nuclear pore protein, influenced the kinetics of NE docking and/or nuclear import. We constructed a HeLa cell line that expresses nucleosomal protein H2B fused to mCherry (H2B-mCherry); H2B-EYFP expressing cells were previously used to study HIV-1 nuclear import [23]. We infected the H2B-mCherry expressing cells (Fig 7A, bottom panel) with HIV-1 virions pseudotyped with VSV-G and compared the kinetics of NE docking and nuclear import to HeLa cells that expressed POM121-mCherry; we did not find any significant differences regarding the time of nuclear import (Fig 7B), time in cytoplasm (Fig 7C), or NE residence time (Fig 7D), indicating that expression of POM121-mCherry did not significantly influence the kinetics of NE docking or nuclear import.

## Discussion

**Fig 8:**
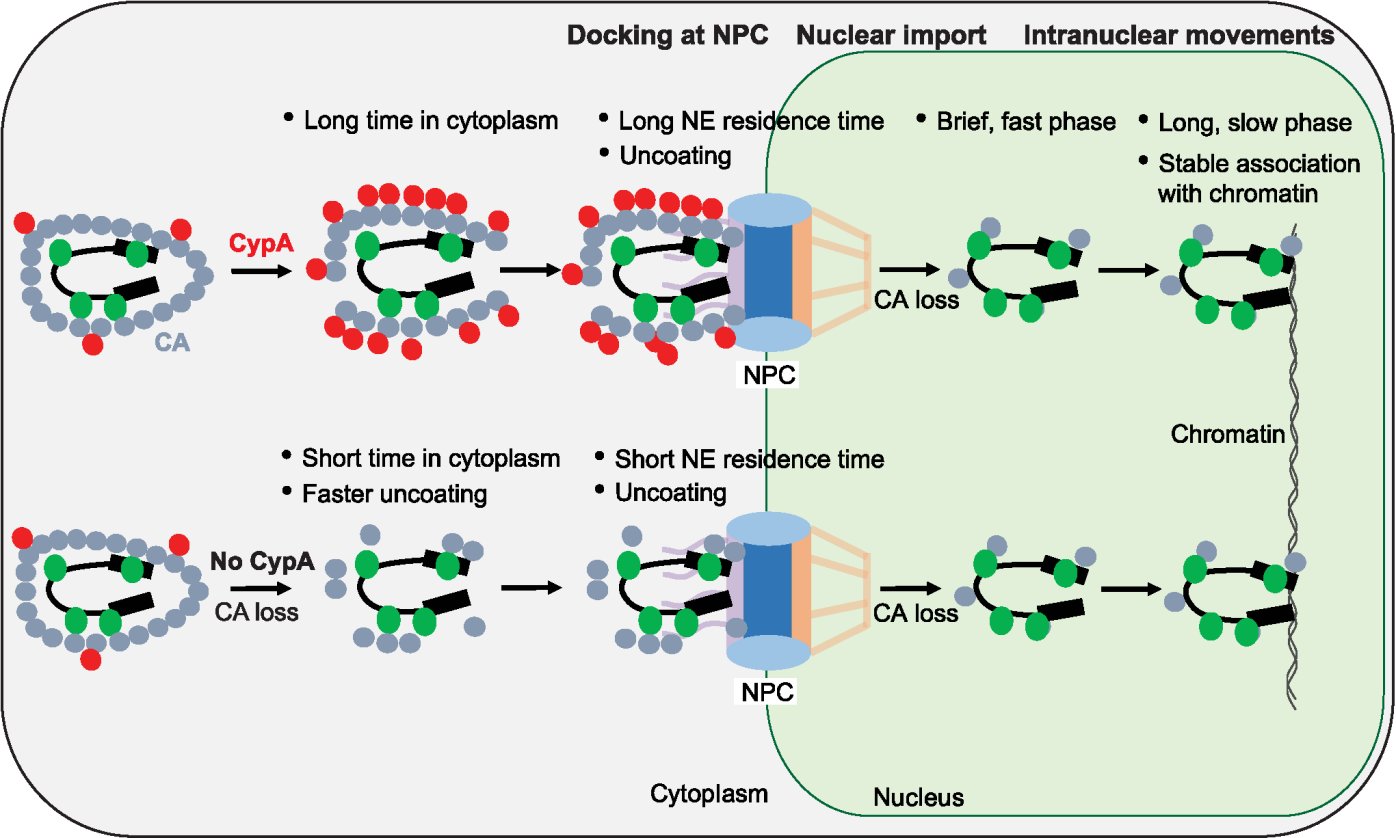
A model for regulation of nuclear import by the CA-CypA interaction and intranuclear movements of viral complexes. The CA-CypA interaction regulates the nuclear import of viral complexes by stabilizing the viral core, which thereby delays the time of docking at the nuclear pore complex (NPC; longer time in cytoplasm) and translocation of viral complexes from the NE to the nucleus (longer NE residence time). Disruption of the CA-CypA interaction results in faster uncoating, NE docking, and nuclear import. After import, the viral complexes exhibit a brief phase of fast mobility as they move away from the nuclear point of entry, followed by a long phase of slow mobility, during which they become tethered to chromatin. Blue circles, CA; red circles, CypA; green circles, A3F-YFP or IN-YFP.

In these studies, we examined the dynamics with which individual HIV-1 complexes formed stable associations with the NE and entered the nucleus, as well as the viral and host factors that regulate these events. A schematic model based on our findings is shown in Fig 8. Briefly, WT HIV-1 complexes are associated with CypA produced in the target cells after fusion, which stabilizes the viral cores, resulting in a longer time in cytoplasm before the viral cores dock at NPCs. After NE docking, the viral cores exhibit a long residence time lasting ∼1.5 hours, before they are imported into the nucleus. Nuclear import of the viral complexes is correlated with substantial loss of CA compared the CA amounts associated with viral complexes at docked at the NE, indicating that one step of viral core uncoating occurs during import. After nuclear import, the viral complexes exhibit a brief fast phase of movement, followed by a long slow phase, during which the viral complexes are hypothesized to tether to chromatin and exhibit movement similar to those of integrated proviruses. When CA-CypA interaction is disrupted by CsA treatment or with the P90A mutation in CA, the viral complexes are associated with less CA in the cytoplasm compared with untreated wild-type viruses, indicating that they undergo faster uncoating, which in turn promotes faster docking at the NPC as well as faster nuclear import. The nuclear viral complexes have similar low levels of CA with and without CsA treatment. Overall, our studies provide valuable insights into essential post-entry events of HIV-1 replication, including the dynamics and regulation of NE docking and nuclear import, and movement of intranuclear viral complexes. Our observations and conclusions regarding these post-entry events are discussed below.

### Docking of viral complexes with the NE and nuclear import

Because nuclear import of HIV-1 has never been observed in living cells, the behavior of the viral complexes at the NE prior to nuclear import is not known. We observed the translocation of 30 A3F-YFP or IN-YFP labeled WT HIV-1 complexes from the cytoplasm to the nucleus, determined the NE residence times of 26 viral complexes, and the intranuclear movements of 21 viral complexes. The behavior of IN-YFP labeled HIV-1 complexes was indistinguishable from the behavior of A3F-YFP labeled viral complexes with respect to their NE residence time, time of import, nuclear penetration distance, and distance traveled from the point of nuclear entry.

Our results provide strong evidence that a long NE residence time prior to import (∼1.5 ± 1.6 hrs) is likely to be a characteristic of viral complexes that are imported and go on to integrate and form a provirus that can express its genome. We also determined that viral complexes cannot enter the nucleus with short NE residence times (<3 min), since nearly all nuclear viral complexes (109/110), with the exception of one dim particle, could be tracked to the NE or to the top or bottom z-slices of the observed nuclear volume. This is in stark contrast to the nuclear import of AAV2 and other large cellular cargos, which dock at the NE and are transported through the nuclear pore within milliseconds [8–10]. Our results show that long and stable associations are necessary but not sufficient for nuclear import and are dependent upon CA and Nup358. The HIV-1 complexes that were imported into the nucleus did not show any apparent lateral movements on the nuclear membrane, suggesting that they were docked at the nuclear pore through which they eventually entered the nucleus.

HIV-1 viral cores are presumably disassembled before nuclear import due to their large size (61-nm width, 120-nm length) compared to the 40-nm size limit for translocation through a nuclear pore [5,6]. The intracellular location at which viral core uncoating occurs has not been fully established. Several studies have suggested that uncoating occurs in the cytoplasm [40,45] and is temporally associated with reverse transcription [62–65], while another study has suggested a role for the cyclophilin homology domain of Nup358 in capsid disassembly at the nuclear pore [48]; however, a direct role for Nup358 in engaging the capsid prior to nuclear import remains controversial [49]. We propose that viral cores may require a long NE residence time, during which they undergo extensive CA dissociation and/or conformational rearrangements that are a prerequisite for nuclear import. Consistent with this hypothesis and in agreement with others [39,66], we observed reduced intensity of CA signal associated with nuclear viral complexes compared to viral complexes docked at the NE, suggesting that one of the viral core uncoating steps occurs during nuclear import. It should also be noted that in addition to viral core uncoating, structural/conformational changes to the NPC may be required to allow nuclear import of HIV-1 complexes.

### Regulation of NE docking and nuclear import

Our results show that the CA-CypA interaction, but not reverse transcription, regulates the dynamics of nuclear import. Disruption of the CA-CypA interaction with CsA treatment or with the P90A mutation resulted in faster nuclear import of viral complexes by decreasing both the time in cytoplasm (faster NE docking) and NE residence time (faster translocation into the nucleus). This result is consistent with a previous report, which concluded that CypA affects nuclear entry of viral cDNA [35]. Although disruption of the CA-CypA interaction reduced the NE residence time from 1.5 hrs to 0.6–0.7 hrs, their NE residence time (∼40 min) is still substantially longer than the milliseconds required for transport of adeno-associated virus or other cellular cargos. Faster nuclear import of viral complexes in CsA-treated cells may be related to the fact their import is insensitive to depletion of nuclear pore proteins Nup153, Nup358 and TNPO3 [32–34].

CsA treatment inhibits HIV-1 replication in some T cell lines and primary CD4^+^ T cells [29–31,35], suggesting that CypA binding evolved to facilitate HIV-1 replication in its natural target cells of infection. Our observation that disrupting the CA-CypA interaction in HeLa cells leads to faster NE docking and nuclear import indicates that one function of CypA binding is to slow down nuclear import. We hypothesize that one function of CypA binding is to protect incompletely synthesized viral DNA from host nucleases and/or DNA repair enzymes in the nucleus, leading to more efficient viral replication. Our hypothesis is consistent with a previous proposal that CypA binding keeps viral nucleic acid sequestered in the viral core and evades innate immune nucleic acid sensors [28]. Development of assays to visualize the nuclear import of HIV-1 in T-cells and monocyte-derived macrophages will be required to test these hypotheses.

Several studies have shown a link between reverse transcription and viral core uncoating and/or conformational changes in the viral core [39,62,63,67]. While these studies clearly showed that reverse transcription triggers loss of CA and/or conformational changes in the viral core, the significance of these structural changes in the core to NE docking or nuclear import was not examined. Interestingly, our results showed that inhibiting reverse transcription did not alter the time in cytoplasm or NE residence time, indicating that the uncoating and/or conformational changes in the viral core that are triggered by reverse transcription are not required for NE docking or nuclear import. We speculate that the changes in the viral core eventually occur in the absence of reverse transcription in a manner that does not affect the kinetics of nuclear import.

### Movement of viral complexes after nuclear import

We observed that right after import the nuclear viral complexes exhibit relatively fast mobility (brief fast phase) followed by a second phase of slower mobility (long slow phase), suggesting that during the brief fast phase viral complexes have not associated with chromatin or large macromolecules and may be diffusing freely. The viral complexes exhibited slower movements in the long slow phase, and their diffusion rate was similar to the rate we observed for HIV-1 transcription sites (∼0.6 × 10^−5^ μm^2^/sec) and the rates previously reported for genes (reviewed in [50]). These observations support the hypothesis that the viral complexes are tethered to chromatin and their movement in the long slow phase is due in large part to the movement of the chromatin. Intriguingly, treatment of target cells with NVP, RAL, or CsA did not affect the mobility of the nuclear viral complexes, indicating that the completion of reverse transcription or successful integration are not required for the proposed tethering to chromatin.

Although LEDGF/p75 is known to interact with HIV-1 IN and target viral complexes to their integration sites, our result suggested that LEDGF/p75 is not the primary determinant of the proposed tethering of viral complexes to chromatin after nuclear import. It is noteworthy that LEDGF/p75 is dispensable for targeting HIV-1 to less condensed euchromatic regions [68]. In future studies, it will be of interest to determine whether CPSF6, a host nuclear protein that is known to bind CA and affect HIV-1 integration [69], HRP-2 [70], or other host proteins that have been reported to interact with integrase [71,72] affect the mobility of HIV-1 nuclear complexes and integration site selection.

Our data support the hypothesis that viral complexes become tethered to chromatin at or near their sites of integration. The peripheral location of viral complexes that we observed is consistent with previous reports indicating that HIV-1 integrates at the nuclear periphery [11,12,73], but in contrast to another report which suggested that integration in wild-type HIV-1 occurs randomly throughout the nucleus [13]. Our observation that the viral complexes may be tethered to chromatin shortly after entering the nucleus and remain near the nuclear periphery is in line with the models proposed by Marini et al. [12], Lelek et al. [73], and Di Primio [11]. However, our data also indicates that the virus-chromatin complex can move away from the nuclear point of entry, albeit slowly, so it may not be correct to assume that a viral complex entered through the nearest nuclear pore.

HIV-1 and other retroviruses have low ratios of infectious to non-infectious particles; consequently, most of the observed viral complexes in microscopy studies may be non-infectious. We previously determined that in our microscopy experiments, ∼50 particles enter each cell, of which ∼2 enter the nucleus, and ∼1 forms a provirus that can express a GFP reporter gene [14]. We determined that the multiplicity of infection (MOI) of A3F-YFP labeled virions in the imaging experiments was ∼1.1, defined as proviruses/cell that express a GFP reporter gene (S1 Table). In the current live-cell microscopy studies, we observed ∼1.6 A3F-YFP labeled viral particles per nucleus (44 A3F-YFP labeled complexes/28 cells), which is close to the MOI of ∼1.1. However, since our A3F-YFP labeling efficiency was ∼50%, we estimate that ∼3.2 viral complexes entered per nucleus, of which ∼1.1 generated a provirus that expressed GFP, indicating that ∼1 of 3 nuclear viral complexes were infectious. Thus, a high percentage of nuclear viral complexes, which may be unlabeled or labeled with A3F-YFP, are infectious. Similar overall behavior of A3F-YFP labeled and IN-YFP labeled viral complexes that entered the nucleus (time in cytoplasm, NE residence time, time of import, nuclear penetration distance, distance from point of entry, and the short fast phase and long slow phase of nuclear movements) suggests that these observed characteristics reflect the average behavior of infectious viral complexes.

In our microscopy studies, only a small proportion of cytoplasmic or NE-associated viral complexes enter the nucleus, indicating that only a minority of cytoplasmic and NE-associated viral complexes are infectious. The amount of CA associated with cytoplasmic and NE-associated viral complexes reflects the average of all viral complexes, and the possibility that this average differs from the amount of CA associated with the infectious viral complexes cannot be excluded. Methods to efficiently and quantitatively detect CA in living cells, such as tetracysteine-tagged CA [74,75], and the recently developed dsRed-tagged CypA [63], are needed to determine the state of the viral cores of infectious viral particles at the NE.

In summary, we determined the kinetics of NE docking of HIV-1 complexes, and showed that the stable associations which involve CA and Nup358 are functionally important for nuclear import. We also observed the translocation of HIV-1 complexes from the cytoplasm to the nucleus and found that the CypA-CA interaction, but not reverse transcription, regulates the timing of nuclear import, by increasing the time the viral complex resides in the cytoplasm as well as at the NE immediately prior to nuclear import. We observed two phases of nuclear movement, suggesting the viral complexes associate with chromatin at or near the site of integration shortly after entering the nucleus. Future studies of the dynamics with which HIV-1 complexes associate with the NE, enter the nucleus, move in the nucleus and interact with chromatin will increase our understanding of these essential steps in HIV-1 replication. Finally, these studies may provide insights into the mechanisms involved in the nuclear import of large cytoplasmic macromolecular complexes.

## Materials and methods

### Cells, reagents, and immunofluorescence staining

HeLa, TZM-bl, and 293T cells were maintained as previously described [14,76]. HeLa cells or TZM-bl cells stably expressing POM121-mCherry were created by transduction with a lentiviral vector containing the human POM121-mCherry expressed from the ubiquitin C promoter. HeLa cells stably expressing H2B-mCherry were created by transduction with a lentiviral vector containing the human H2B-mCherry expressed from the CMV promoter (Addgene plasmid #20972; [77]). Immunofluorescence staining was performed as previously described [14]; AG3.0 antibody was used to detect HIV-1 CA (NIH AIDS Reagent Program). NVP and RAL were obtained through the NIH AIDS Reagent Program and was used at a final concentration of 5 μM and 10 μM, respectively. CsA (Millipore) was used at a final concentration of 5 μM. Cell viability was determined using the ATPlite Luminescence Assay System (PerkinElmer) according to manufacturer’s protocol.

### siRNA-mediated knockdown of host gene expression

HeLa cells were reverse-transfected with siRNA targeting Nup358 or control siRNA (Ambion) using RNAiMax (Invitrogen) as previously described [14].

### Western blot analysis

Cell lysates were harvested and analyzed by sodium dodecyl sulfate-polyacrylamide gel electrophoresis followed by Western Blot analysis using the Odyssey System (Li-Cor) as previously described [78]. Antibodies were obtained for specific detection of Nup358 (Abcam), LEDGF/p75 (Cell Signaling), or α-tubulin (Sigma). Primary antibodies were detected using either infrared dye-labeled (800C) goat anti-rabbit or infrared dye-labeled (680) goat anti-mouse secondary antibodies (Li-Cor).

### Generation of a LEDGF/p75 knockout cell line

HeLa cells were transfected (TransIT-LT1 Transfection Reagent; Mirus Bio LLC) with 0.5 μg each of Cas9-GFP (Addgene #44719) plus plasmids expressing gRNA LFor (5’- GGCTAAGTATAATGAATTAG -3’) and gRNA LRev (5’- TTTAGAACATGTTCTTGGT-3’). The gRNAs were positioned to bind in intron 11 and exon 14 of LEDGF to delete the IBD domain (Fig 4D). Single cell clones were isolated by limiting dilution. Genomic DNA isolated from single cell clones was amplified by PCR using primers outside the gRNA binding sites (Lfor 5’-GGGACTGGAGAGCAGAGGAGTTATC-3’ and Lrev 5’- GCTCTAGTCCTTCAATAGGCCCATCAGA-3’), producing either undeleted (1158 bp) or deleted IBD bands (∼500 bp). Clones positive for deleted IBD domains were further sequenced (Macrogen) to verify Cas9/gRNA cleavages. Cell lysates from positive clones were analyzed by Western Blot analysis (described above).

### Plasmids, virus production, and infection

Fluorescent protein-labeled HIV-1 particles were prepared by co-transfection of 293T cells with the HIV-1-derived vector pHDV-EGFP (10 μg; [79]) or pH-EGFP (10μg; pH-EGFP is derived from pHL [80] except that vif and vpr were deleted and EGFP replaced the luciferase gene), HCMV-G (2.0 μg, VSV-G; [81]), and A3F-YFP (1.25 μg) or Vpr-IN-YFP (5.0 μg) as previously described [14]. In some experiments, an HIV-1 envelope expression plasmid (AD8; 2.0 μg) was used instead of HCMV-G [82]. Vpr-IN-YFP is similar to Vpr-IN-GFP [23], except that GFP was replaced with YFP and the HIV-1 protease cleavage site between Vpr and IN (IRKVL/FLDGI) is preceded by a flexible glycine-rich linker (“/” indicates protease cleavage). In some experiments, the viral membrane marker S15-mCherry (5 μg) was also used [44]. In some experiments, the CA region in HDV-EGFP was replaced with CA mutants K203A and E128A/R132A [14]. The CA region in pH-EGFP was replaced with CA mutant P90A, which was created using site-directed mutagenesis (Agilent). For additional virus characterization, a 1:1 mixture of unlabeled Gag (pHDV-EGFP) and Gag-iCFP (pGag-iCFP; contains a CFP between MA and CA and is flanked by HIV-1 protease cleavage sites [kindly provided by Dr. Marc Johnson, University of Missouri]) was used during virus production so that HIV-1 particles could be identified by CFP fluorescence. pC-Help (an HIV-1 helper construct that lacks several cis-acting elements needed for viral replication, including the packaging signal and primer-binding site), GagCeFP-BglSL (HIV-1 construct containing 18 copies of Bgl stem loops), and BglG-YFP expression vector were previously described [83]. HIV-1 particles containing the genomes with Bgl stem loops were prepared by co-transfection of 293T cells with GagCeFP-BglSL (8 μg), pC-help (3 μg), and HCMV-G (2.0 μg, VSV-G).

HeLa cells were seeded in ibiTreated μ-slides (3 × 10^4^ cells/well) one day prior to infection. Cells were infected with a normalized number of fluorescently-labeled particles via spinoculation at 16°C, which permitted virion binding to cell membranes but prevented virion endocytosis as previously described [14,84]. After centrifugation, the media was replaced with prewarmed media to allow internalization of the virus (defined as the 0-h time point) and thereafter incubated at 37°C. Infections with A3F-YFP labeled virions were performed at a multiplicity of infection (MOI) of ∼1 (S1 Table). Time-lapse images of the infected cells were acquired by epifluorescence microscopy (described below) or the cells were fixed at various time points post-infection with 4.0% (wt/vol) paraformaldehyde (PFA) and imaged by confocal microscopy (described below). To determine infectivity for the viruses containing a GFP reporter gene (viruses made using pHDV-EGFP), the percentage of GFP^+^ cells was determined by flow cytometry analyses performed on a FACSCalibur system (BD Biosciences) 48 hrs post-infection.

### Confocal microscopy and image processing

Confocal images were acquired of fixed cells using an LSM710 laser scanning confocal microscope (Zeiss) with a Plan-Apochromat 63x N.A.-1.40 oil objective, using 405-nm, 515-nm, 561-nm, and 633-nm lasers for illumination or a Nikon Eclipse T*i*-E microscope equipped with a Yokogawa CSU-X1 spinning disk unit with a Plan-Apochromat 60x N.A. 1.40 oil objective, using 405-nm, 514-nm, and 633-nm lasers for illumination. The diffraction-limited spots were detected and their positions were determined in each image using Localize [85]. The positions of the spots were used to determine colocalization; spots were considered colocalized if the centers of the spots were within 3 pixels. Colocalization of the YFP particles with the NE mask (based on Lamin A/C immunofluorescence staining) was determined using a custom-written MATLAB program (Mathworks). A mask of the nucleus interior was also created using the NE mask. The percentage of cytoplasmic YFP particles that colocalized with the NE mask and the percentage of YFP particles that colocalized with the nucleus mask were determined.

A custom-written MATLAB program was used to determine the colocalization of A3F-YFP with HIV-1 p24 capsid (CA) signal in the cytoplasm, at the NE, and inside the nucleus of infected cells. Because of variable background intensity across different regions of the cell, the intensity values were determined in the Cy5 channel (channel used to detect CA) at random positions in the cytoplasm, at the NE, and inside the nucleus. The threshold intensity values were determined as the mean + 1 SD of the random intensities for each region. The intensity values of the Cy5 channel at the position of each A3F-YFP particle was determined; A3F-YFP particles co-localizing with Cy5 signals that were above the threshold intensity value were considered positive for CA.

### Epifluorescence microscopy

Epifluorescence microscopy was performed as previously described, with some modifications [51]. Briefly, we employed an inverted Nikon Eclipse Ti microscope and a 100 × 1.45-N.A. oil objective, using 514-nm and 594-nm lasers for illumination. A 1x tube lens (pixel size = 0.160 μm) or a 1.5x tube lens (pixel size = 0.107 μm) was used. Digital images were acquired using an Andor iXon3 897 Camera and NIS-Elements software (Nikon) with emission filters of 542/27 nm and 650/75 nm, respectively. Simultaneous dual-color imaging was performed by using a Four-Channel Simultaneous-Imaging System (QV2; Photometrics); the separate channels were manually aligned using fluorescent virus particles on a slide prior to each experiment. In addition, the images of separate channels were further aligned with single-pixel accuracy using a custom-written MATLAB program.

The localization precision of the microscope was determined by acquiring time-lapse images of immobilized A3F-YFP labeled virus particles on a slide under the same conditions as live-cell imaging experiments. Single-particle tracking of A3F-YFP particles (described below) was performed and the single-step jump distances (i.e. the distance a particle moves between two consecutive frames) were determined. The average 1-step jump distance for all YFP (∼43 nm) signals indicates a localization precision for each channel that is suitable for single-particle tracking.

### Colocalization of HIV-1 complexes with the NE in living cells

Virus particle movement was followed by acquiring time-lapse images at 9.8 Hz with a 100-ms integration time and ∼2-ms overhead between frames, resulting in an overall 102-ms frame time. Since high frame rates were used to capture viral movements, we were limited to short, 1-min movies due to photobleaching. Therefore, several 1-min movies were taken of the infected cells (1 movie per cell) from 45 mpi to 2 hpi. The focal plane was selected at approximately equatorial plane of the cells. It was not necessary to deconvolve images because contribution of out-of-focus light was minimal (signal-to-noise ratio >4). Single-particle tracking was performed with MATLAB code based on the available tracking algorithms [86], with maximum single-step displacement of five pixels (0.54 μm) when using the 1.5x tube lens and a minimum track length of five consecutive frames. The positions of the diffraction-limited spots in the tracks were refined with 2D Gaussian fit [87].

Colocalization of the A3F-YFP tracks with the POM121-mCherry signal was determined using a custom-written MATLAB program. First, it was determined that there is no detectable cell movement within the 1-min movies (S1A Fig; S1 Movie). Therefore, a mask of the NE using the POM121-mCherry signal was created using an average projection of the mCherry channel of the entire time series. This mask was approximately 1 μm in width and was centered on the POM121-mCherry signal (Fig 2B). Occasionally, there were regions of the NE in which the POM121-mCherry signal was not well-defined and therefore a mask could not be accurately created. These ill-defined regions were manually selected and any tracks that entered these regions were excluded from the analyses. The longest consecutive residence time for each A3F-YFP particle that colocalized with the POM121 mask for at least 1 frame was determined.

To determine the kinetics of association of randomly moving particles with the NE, a simulation was performed using a custom-written MATLAB program. Briefly, a mask of the nucleus was created using the NE mask (described above). The masks were made using the POM121-mCherry signal obtained during a typical experiment. Then, a simulation of randomly moving particles was performed with 2 parameters; particles could not leave the outer limits of the image and could not colocalize with the nucleus mask (since in a typical 1-min movie we did not observe translocation of HIV-1 complexes from the NE to the nucleus). For each experiment, the random movement of particles around 3 different nuclei was simulated, resulting in ∼550 particles that contacted each NE mask during the 1-min simulation (>1600 total particles). Next, the longest consecutive residence time for each particle that contacted the NE mask for at least 1 frame was determined. The same random movement simulation was repeated two more times and analyzed.

To visualize the association of HIV-1 complexes with the NE over a longer time period, a 9-slice z-stack (taken at 0.4-μm step intervals) was acquired every 1 min for 2 hrs starting at 10 mpi to 6 hpi. Each z-stack, which covered an axial depth of ∼4 μm, was centered near the equatorial plane of the nucleus; the top and bottom of the nuclei were not imaged to minimize photobleaching. The residence time for each particle that was associated with the NE for 2 consecutive frames (1 min) was determined manually.

### Visualization of nuclear import

To observe nuclear import of A3F-YFP-labeled or IN-YFP-labeled particles, a 9-slice z-stack (taken at 0.4-μm step intervals) was acquired every 3 min shortly after infection for up to 10 hrs. Each z-stack, which covered an axial depth of ∼4 μm, was centered near the equatorial plane of the nucleus; the top and bottom of the nuclei were not imaged to minimize photobleaching during the long movies (S3A Fig). As a result, particles that entered the nucleus from the top or bottom were not detected. Deconvolution was applied to the images to remove the out of focus noise using Huygens software (SVI, Netherlands). The lateral movement of the nucleus during each movie was corrected using the POM121-mCherry signal and a custom-written MATLAB program (S5 Movie). After adjusting for the movement of the nucleus during the time-course, 3D localization (x,y,z) of the YFP-labeled particles was conducted using FISH-QUANT software [88]. U-track algorithm was used for 3D single-particle tracking [89].

### Determination of mean square displacements (MSDs) and diffusion coefficients

Ensemble MSDs were calculated from positional coordinates as previously described [90]. In free diffusion, the MSDs [r^2^(t)] for 3D tracks are linearly related to time (t) and diffusion coefficient (D) by the formula r^2^(t) = 6Dt. The MSDs for 2D tracks are linearly related to time and diffusion coefficient by the formula r^2^(t) = 4Dt. The diffusion coefficients were calculated using the first four time lags.

### Determination of distance between NE and nuclear PICs

The nuclear penetration distance of the HIV-1 complexes imported into the nuclei was determined using a custom-written MATLAB program. A mask of the movement-corrected nucleus (POM121-mCherry images; described above) was created for each time-point. Then, a series a 2-pixel wide (0.32 μm) concentric rings were created; the particle positions (as determined by 3D single-particle tracking) were used to determine the colocalization with the nuclear rings (S3C Fig). For each HIV-1 complex that we observed enter the nucleus, the average nuclear penetration distance (calculated by averaging the nuclear penetration distance for all frames in which the HIV-1 complex was located in the nucleus), maximum nuclear penetration distance, average distance from the nuclear point of entry (calculated by averaging the distances between each of the positions of the HIV-1 complex inside the nucleus and the position of the HIV-1 complex on the NE one frame before nuclear import), maximum distance from the nuclear point of entry, observation time at the nuclear envelope and inside the nucleus, time of NE association (relative to time of infection), and time of nuclear import (relative to time of infection) were determined.

## Supporting information

S1 Table Determination of multiplicity of infection for live-cell microscopy experiments. ^1^ p24 CA amount was determined by ELISA. Values represent the average ± SD of three virus preparations. ^2^ Cells were challenged with a low amount of A3F-YFP labeled HIV-1 GFP-reporter virus so that infectivity could be accurately determined. The percentage of GFP^+^ cells was determined by flow cytometry 48 hrs after infection. ^3^ The multiplicity of infection (MOI) is defined here as the estimated number of GFP-expressing proviruses/cell. ^4^ The MOI for the live-cell microscopy experiments was estimated by dividing the p24 CA amount used for live-cell microscopy experiments by the p24 CA amount used to determine infectivity, and then multiplying this number by the measured infectivity (6.0/0.4 x 7.3 = ∼1.10). ^5^ The number of A3F-YFP labeled viral complexes in each nucleus was determined from the movies used to visualize nuclear import; we observed a total of 44 A3F-YFP labeled nuclear particles in 28 cells. ^6^ The virion labeling efficiency with A3F-YFP was ∼50% (S4C Fig); therefore, an equal number of unlabeled nuclear viral complexes is expected. ^7^ The estimated number of viral complexes/nucleus includes A3F-YFP labeled and unlabeled viral complexes.
(DOCX)

S2 Table Dynamics of A3F-YFP- and IN-YFP-labeled HIV-1 complexes at the NE and after nuclear import. ^1^ A total of 21 HIV-1 complexes were automatically tracked after correction for nucleus movement (7 A3F-YFP labeled complexes [particles 1–7] and 14 IN-YFP labeled complexes [particles 11–24]), which are included in Figs 3 and 4. Nine HIV-1 complexes were detected manually from additional movies (3 A3F-YFP labeled complexes [particles 8–10] and 6 IN-YFP complexes [particles 25–30]) to determine time in cytoplasm, NE residence time, and time of nuclear import. ^2^ No significant differences between the nuclear penetration distance, distance from point of nuclear entry, time in cytoplasm, NE residence time, observation time in nucleus, and time of nuclear import for A3F-YFP and IN-YFP complexes were observed (*P* > 0.05, *t*-test or Mann Whitney test); average values for both A3F-YFP and IN-YFP complexes are shown. ^3^ The time in cytoplasm represents the length of time between the time of infection and the time each viral complex arrived at the NE (see Fig 6A). ^4^ Four A3F-YFP labeled complexes were at the NE prior to beginning of movie (particles 4–7); therefore, the time in cytoplasm is an overestimate and the NE residence time is an underestimate for these complexes. The time in cytoplasm, NE residence time, and time of nuclear import for these particles was not included in the average values. The time in cytoplasm, NE residence time, and time of nuclear import for all particles except particles 4–7 (26 total) were included in Table 1 and Figs 6 and 7. ^5^ Nuclear HIV-1 complexes were observed until the end of the movie or until they exited the z-stack. ^6^ hpi, hours post-infection.
(DOCX)

S1 Fig Effect of Nup358 knockdown on NE association and nuclear import of A3F-YFP labeled HIV-1 complexes. **(A)** RNAi-mediated knockdown of Nup358 in HeLa cells. Cell lysates were collected ∼48-hrs after transection of control or Nup358 siRNA and analyzed by Western blot using antibodies targeting Nup358 or α-tubulin. The numbers below blot indicate the percent Nup358 band intensity compared to the control siRNA. **(B)** The percentage of cytoplasmic A3F-YFP particles that are at the NE and **(C)** the percentage of total A3F-YFP particles in the nucleus at 6 hpi. HeLa cells were transfected with control or Nup358 siRNA and then infected with VSV-G-pseudotyped HIV-1 particles labeled with A3F-YFP ∼48 hrs later. The infected cells were fixed at 6 hpi, the NE was detected by immunofluorescence staining for Lamin A/C, and then visualized by confocal microscopy. Error bars indicate the SD of four experiments. An average of ∼500 A3F-YFP particles from ∼115 cells were analyzed in each experiment. *, *P* ≤ 0.05, *t* test. **(D)** Cell viability after siRNA knockdown of Nup358. HeLa cells were transfected with control or Nup358 siRNA and then analyzed for cell viability using the ATPlite assay at a time when imaging experiments were performed, ∼48 hrs after siRNA transfection. Error bars indicate the SD of three experiments; n.s., not significant (*P* > 0.05; *t*-test).
(EPS)

S2 Fig Analysis of transient associations of HIV-1 complexes with the NE. (**A**) Experimental protocol. HeLa cells stably expressing POM121-mCherry were infected with HIV-1 labeled with A3F-YFP. One-minute long movies were acquired for each infected cell at 10 frames/sec and were initiated 45–120 min post-infection (1 movie/cell). **(B)** A mask of the NE (red) that extends ∼0.5 μm towards the cytoplasm was created using the POM121-mCherry signal (grey). Tracks that colocalized with ill-defined areas of the NE (blue rectangle) were not analyzed. The right panel shows an enlarged image of the white square in the left panel. Scale bar, 5 μm (left), 1 μm (right). **(C)** Determination of cell movement during 1-min movies. An overlay of the first (red) and last (green) frames from a time-lapse movie for the POM121-mCherry channel indicates no significant cell movement during the 1-min movies (see S1 Movie). Scale bar, 5 μm (left), 1 μm (right). **(D)** Simulation of random particle movement near the POM121-mCherry mask. A simulation was performed using a custom-written MATLAB program (as described in Materials and Methods) to determine the kinetics of association of randomly moving particles with the POM121-mCherry mask. The longest consecutive residence time for each particle that contacted the POM121-mCherry mask for at least 1 frame (0.1 sec) was determined. The percentage of particles that colocalized with the POM121-mCherry mask for the indicated minimum NE residence time is plotted using 5 sec bins. For example, 0.9% of the particles colocalized with the POM121-mCherry mask for ≥ 5 sec. Error bars represent the SD of 3 independent simulations. **(E)** Examples of HIV-1 complexes (green) that make transient associations with the NE (≥ 5 sec– 1 min) or < 5 sec contacts with the NE, as defined by the POM121 mask (grey). The YFP tracks (bottom panels) and the maximum intensity projections for the YFP (green) and mCherry (red) channels (top panels) are shown (S2–S4 Movies); white number, length (sec) of colocalization with the POM121 mask (gray). Black numbers indicate length of tracks. Scale bar, 1 μm. (**F-G**) Effect of CA stability mutations and Nup358 knockdown on the NE residence time for A3F-YFP labeled HIV-1 complexes. **(F)** The A3F-YFP tracks from HIV-1 complexes containing WT, K203A, or E128A/R132A capsid mutations (left panel) or after treatment of cells with control or Nup358 siRNA (right panel) that colocalized with the POM121-mCherry mask for at least one frame (0.1 sec) were analyzed for their longest consecutive residence time at the POM121-mCherry mask. The percentage of total particles that colocalized with the POM121-mCherry mask for the indicated minimum NE residence time is plotted using 5 sec bins. The numbers in parentheses to the right of each sample name indicate the number of tracks (compiled from 3–5 independent experiments) that contacted the POM121-mCherry mask for at least 1 frame and were analyzed. **(G)** The percentage of A3F-YFP labeled viral complexes described in **(F)** that colocalized with the POM121 mask and remained for ≥ 5 sec; >20 movies (1 cell per movie) were analyzed for each condition and experiment. Error bars indicate SD of 3–5 experiments.
(EPS)

S3 Fig Three-dimensional (3D) tracking of HIV-1 complexes. **(A)** A 3D reconstruction of the first frame after nuclear import for the A3F-YFP particle (green) and POM121-mCherry signal (red) described in Fig 3A and 3B. Arrow indicates A3F-YFP signal inside nucleus. The X, Y, and Z axes of the images are indicated. **(B)** A 3D track is shown for the A3F-YFP particle described in Fig 3A and 3B. Arrow indicates the first frame in which the HIV-1 complex was observed in the nucleus. **(C)** Determination of nuclear penetration distance of HIV-1 complexes. To determine the nuclear penetration distance for an HIV-1 complex, a series of ∼2-pixel wide (0.32 μm) rings (visualized as alternating white and black rings) were created in the nucleus (as determined by the POM121-mCherry signal; red) for each time point as described in Material and Methods. The positions of the HIV-1 complex in each frame (as determined by 3D single-particle tracking) were used to determine colocalization with the nuclear rings. Scale bar, 5 μm.
(EPS)

S4 Fig Relative infectivity, single virion analysis and detection of HIV-1 complexes in infected cells by A3F-YFP and IN-YFP. **(A)** Relative infectivity of HIV-1 without any label (Control) or HIV-1 labeled with IN-YFP. **(B)** Examples of individual HIV-1 virions (Gag-iCFP^+^) labeled with A3F-YFP (top row) or IN-YFP (bottom row). Scale bar, 5 μm. **(C)** The percentage of Gag-iCFP^+^ particles that are YFP^+^. **(D)** The average YFP particle intensity for Gag-CFP^+^/YFP^+^ particles; a.u., arbitrary units. Error bars indicate SD of 3 experiments. **(E-G)** HeLa cells were infected with VSV-G-pseudotyped HIV-1 labeled with A3F-YFP or with IN-YFP. The infected cells were fixed at various time points post-infection, the NE was detected by immunofluorescence staining for Lamin A/C, and then visualized by confocal microscopy. The number of YFP particles/cell **(E)**, the percentage of cytoplasmic YFP particles that are at the NE **(F)**, and the percentage of YFP particles in the nucleus **(G)** were determined at various time points post-infection. An average of 1814, 775, 584 and 513 YFP particles from 94 cells were analyzed for the 0, 1, 3, and 6 hr time points, respectively. Error bars indicate the SD of six experiments. n.s., not significant (*P* > 0.05, *t*-test).
(EPS)

S5 Fig Sequence verification of HLKO clone. Two allele patterns were identified: Allele 1 shows an inversion (black line) + 2 bp insertion (black box) between the two gRNA binding sites (yielding an undeleted 1160 bp band as seen in Fig 5B), and Allele 2 shows a 678-bp deletion between the two gRNA binding sites (yielding a ∼480-bp deleted band as seen in Fig 5B). Binding sites for each gRNA are boxed in red (a and b); PAM sequences are shown in bold.
(EPS)

S6 Fig Effect of disruption of CA-CypA interaction on the efficiency of nuclear import and HIV-1 infectivity after CsA treatment or with a CypA-binding CA mutant. **(A)** The percentage of total A3F-YFP labeled HIV-1 complexes in the nucleus at 2, 6, and 24 hpi with WT CA in untreated cells (WT), WT CA in cells treated with CsA (WT+CsA), and P90A CA mutant virus in untreated cells (P90A). HeLa cells were infected with VSV-G-pseudotyped HIV-1 particles labeled with A3F-YFP in the presence or absence of CsA. The infected cells were fixed at 2, 6, and 24 hpi, the NE was detected by immunofluorescence staining for Lamin A/C, and then visualized by confocal microscopy. An average of ∼850 A3F-YFP particles from ∼100 cells were analyzed for each condition. **(B)** Relative infectivity of an HIV-1 GFP-reporter virus with WT CA in untreated cells (WT), WT CA in cells treated with CsA (WT+CsA), and P90A CA mutant virus (P90A). The percentage of GFP+ cells was determined by flow cytometry 48 hrs after infection (WT set to 100%). Error bars indicate the SD of at least 3 experiments. *, *P* ≤ 0.05, *t*-test; n.s., not significant (*P* > 0.05), *t*-test.
(EPS)

S1 Movie POM121-mCherry signal in a cell during a typical 1-min movie. The movie is comprised of 592 frames. Scale bar, 5 μm. Time-lapse images were acquired at 9.8 Hz with a 100-ms integration time. The movie is encoded at 30 Hz (∼3x real-time). Time scale, seconds; Scale bar, 1 μm.
(AVI)

S2 Movie An A3F-YFP labeled HIV-1 complex that forms a semi-stable association with the POM121-mCherry mask. The movie is comprised of 590 frames. Time-lapse images were acquired at 9.8 Hz with a 100-ms integration time. A Laplacian of Gaussian filter was applied to the images. The movie is encoded at 30 Hz (∼3x real-time). The trajectory is indicated by changing colors from start (blue) to finish (red). Time scale, seconds; Scale bar, 1 μm.
(AVI)

S3 Movie An A3F-YFP labeled HIV-1 complex that forms a semi-stable association with the POM121-mCherry mask. The movie is comprised of 177 frames. Time-lapse images were acquired at 9.8 Hz with a 100-ms integration time. A Laplacian of Gaussian filter was applied to the images. The movie is encoded at 30 Hz (∼3x real-time). The trajectory is indicated by changing colors from start (blue) to finish (red). Time scale, seconds; Scale bar, 1 μm.
(AVI)

S4 Movie An A3F-YFP labeled HIV-1 complex that is forms a transient association with the POM121-mCherry mask. The movie is comprised of 70 frames. Time-lapse images were acquired at 9.8 Hz with a 100-ms integration time. A Laplacian of Gaussian filter was applied to the images. The movie is encoded at 30 Hz (∼3x real-time). The trajectory is indicated by changing colors from start (blue) to finish (red). Time scale, seconds; Scale bar, 1 μm.
(AVI)

S5 Movie The movement of the nucleus (POM121-mCherry signal) during 10 hrs. A comparison of the unadjusted (left) and adjusted (right) movement of a nucleus is shown. The movement of the nucleus was adjusted relative to the position of the nucleus in the first frame as described in Materials and Methods. The movie is encoded at 10 Hz (∼1800x real-time) is comprised of 201 frames. Time scale, hours:minutes; Scale bar, 10 μm.
(AVI)

S6 Movie The translocation of an A3F-YFP labeled HIV-1 complex that was stably associated with the NE for ∼20 min before entering the nucleus. A 9-slice z-stack (0.4-μm interval) was acquired every 3 min for 10 hrs starting at 45 mpi. For each frame, an average intensity projection using the z-slice in which the A3F-YFP particle of interest was located (as determined by 3D particle tracking) and the z-slices immediately above and below for each time point was performed for each channel (A3F-YFP, green; POM121-mCherry, red) to enhance signal-to-noise ratio. The movement of the nucleus was adjusted relative to the position of the nucleus in the first frame as described in the Methods. The movie is encoded at 10 Hz (∼1800x real-time) and is comprised of 108 frames. Time scale, hours:minutes; Scale bar, 5 μm.
(AVI)

S7 Movie The translocation of an A3F-YFP labeled HIV-1 complex that was stably associated with the NE for > 3 hrs before entering the nucleus. A 9-slice z-stack (0.4 μm interval) was acquired every 3 min for 10 hrs starting at 1 hpi. For each frame, an average intensity projection using the z-slice in which the A3F-YFP particle of interest was located (as determined by 3D particle tracking) and the z-slices immediately above and below for each time point was performed for each channel (A3F-YFP, green; POM121-mCherry, red) to enhance signal-to-noise ratio. The movement of the nucleus was adjusted relative to the position of the nucleus in the first frame as described in Materials and Methods (S5 Movie). The movie is encoded at 10 Hz (∼1800x real-time) and is comprised of 201 frames. Time scale, hours:minutes; Scale bar, 5 μm.
(AVI)

S8 Movie The translocation of an IN-YFP labeled HIV-1 complex that was stably associated with the NE for 1.5 hrs before entering the nucleus. A 9-slice z-stack (0.4 μm interval) was acquired every 3 min for 10 hrs starting at 10 mpi. For each frame, an average intensity projection using the z-slice in which the IN-YFP particle of interest was located (as determined by 3D particle tracking) and the z-slices immediately above and below for each time point was performed for each channel (IN-YFP, green; POM121-mCherry, red) to enhance signal-to-noise ratio. The movement of the nucleus was adjusted relative to the position of the nucleus in the first frame as described in Materials and Methods. The movie is encoded at 10 Hz (∼1800x real-time) and is comprised of 118 frames. Time scale, hours:minutes; Scale bar, 5 μm.
(AVI)
