# Supplementary material for: Dynamics and regulation of nuclear import and nuclear movements of HIV-1 complexes
Source: PLoS Pathog. 2017 Aug 21;13(8):e1006570. doi: 10.1371/journal.ppat.1006570 (PMC5578721; doi:10.1371/journal.ppat.1006570)
Supplement: S1 Table — 1 p24 CA amount was determined by ELISA. Values represent the average ± SD of three virus preparations. 2 Cells were challenged with a low amount of A3F-YFP labeled HIV-1 GFP-reporter virus so that infectivity could be accurately determined. The percentage of GFP+ cells was determined by flow cytometry 48 hrs after infection. 3 The multiplicity of infection (MOI) is defined here as the estimated number of GFP-expressing proviruses/cell. 4 The MOI for the live-cell microscopy experiments was estimated by dividing the p24 CA amount used for live-cell microscopy experiments by the p24 CA amount used to determine infectivity, and then multiplying this number by the measured infectivity (6.0/0.4 x 7.3 = ∼1.10). 5 The number of A3F-YFP labeled viral complexes in each nucleus was determined from the movies used to visualize nuclear import; we observed a total of 44 A3F-YFP labeled nuclear particles in 28 cells. 6 The virion labeling efficiency with A3F-YFP was ∼50% (S4C Fig); therefore, an equal number of unlabeled nuclear viral complexes is expected. 7 The estimated number of viral complexes/nucleus includes A3F-YFP labeled and unlabeled viral complexes. (DOCX) [file ppat.1006570.s001.docx]

**S1 Table. Determination of multiplicity of infection for live-cell microscopy experiments.**

| **Experiment Type** | **Label** | **p24 CA**  **(ng)** ^1^ | **Infectivity (%GFP cells)^2^** | **Multiplicity of infection**^3^ | **Number of**  **A3F-YFP viral complexes/nucleus**^5^ | **Estimate number of unlabeled viral complexes/nucleus^6^** | **Estimated number of viral complexes/nucleus^7^** |
| --- | --- | --- | --- | --- | --- | --- | --- |
| FACS analysis | A3F-YFP | 0.4 ± 0.1 | 7.3 ± 3.2 | 0.07 | - | - | - |
| Live-cell microscopy | A3F-YFP | 6.0 ± 1.9 | - | 1.10^4^ | 1.6 | 1.6 | 3.2 |

^1^ p24 CA amount was determined by ELISA. Values represent the average ± SD of three virus preparations.

^2^ Cells were challenged with a low amount of A3F-YFP labeled HIV-1 GFP-reporter virus so that infectivity could be accurately determined. The percentage of GFP^+^ cells was determined by flow cytometry 48 hrs after infection.

**^3^** The multiplicity of infection (MOI) is defined here as the estimated number of GFP-expressing proviruses/cell.

^4^ The MOI for the live-cell microscopy experiments was estimated by dividing the p24 CA amount used for live-cell microscopy experiments by the p24 CA amount used to determine infectivity, and then multiplying this number by the measured infectivity (6.0/0.4 x 7.3 = ~1.10).

^5^ The number of A3F-YFP labeled viral complexes in each nucleus was determined from the movies used to visualize nuclear import; we observed a total of 44 A3F-YFP labeled nuclear particles in 28 cells.

^6^ The virion labeling efficiency with A3F-YFP was ~50% (S4C Fig); therefore, an equal number of unlabeled nuclear viral complexes is expected.

^7^ The estimated number of viral complexes/nucleus includes A3F-YFP labeled and unlabeled viral complexes.
