# Supplementary material for: Dynamics and regulation of nuclear import and nuclear movements of HIV-1 complexes
Source: PLoS Pathog. 2017 Aug 21;13(8):e1006570. doi: 10.1371/journal.ppat.1006570 (PMC5578721; doi:10.1371/journal.ppat.1006570)
Supplement: S2 Table — 1 A total of 21 HIV-1 complexes were automatically tracked after correction for nucleus movement (7 A3F-YFP labeled complexes [particles 1–7] and 14 IN-YFP labeled complexes [particles 11–24]), which are included in Figs 3 and 4. Nine HIV-1 complexes were detected manually from additional movies (3 A3F-YFP labeled complexes [particles 8–10] and 6 IN-YFP complexes [particles 25–30]) to determine time in cytoplasm, NE residence time, and time of nuclear import. 2 No significant differences between the nuclear penetration distance, distance from point of nuclear entry, time in cytoplasm, NE residence time, observation time in nucleus, and time of nuclear import for A3F-YFP and IN-YFP complexes were observed (P > 0.05, t-test or Mann Whitney test); average values for both A3F-YFP and IN-YFP complexes are shown. 3 The time in cytoplasm represents the length of time between the time of infection and the time each viral complex arrived at the NE (see Fig 6A). 4 Four A3F-YFP labeled complexes were at the NE prior to beginning of movie (particles 4–7); therefore, the time in cytoplasm is an overestimate and the NE residence time is an underestimate for these complexes. The time in cytoplasm, NE residence time, and time of nuclear import for these particles was not included in the average values. The time in cytoplasm, NE residence time, and time of nuclear import for all particles except particles 4–7 (26 total) were included in Table 1 and Figs 6 and 7. 5 Nuclear HIV-1 complexes were observed until the end of the movie or until they exited the z-stack. 6 hpi, hours post-infection. (DOCX) [file ppat.1006570.s002.docx]

**S2 Table. Dynamics of A3F-YFP- and IN-YFP-labeled HIV-1 complexes at the NE and after nuclear import**

| **Particle^1^** | **Label** | **Nuclear penetration distance (µm)^2^** | | **Distance from point of entry (µm)^2^** | | **Time in cytoplasm (hours)^2,3^** | **NE residence time (hours)^2^** | **Observation time in nucleus (hours)^2,5^** | **Time of nuclear import (hpi)^2,6^** |
| --- | --- | --- | --- | --- | --- | --- | --- | --- | --- |
|  |  | **Avg. ± SD** | **Max.** | **Avg. ± SD** | **Max.** |  |  |  |  |
| 1 | A3F-YFP | 1.8±0.6 | 3.2 | 3.2±0.9 | 4.9 | 5.2 | 0.6 | 5.3 | 5.8 |
| 2 | A3F-YFP | 1.5±0.5 | 2.2 | 2.5±0.6 | 3.9 | 2.1 | 0.4 | 5.9 | 2.4 |
| 3 | A3F-YFP | 2.0±0.6 | 2.9 | 3.0±0.8 | 3.9 | 2.1 | 0.7 | 2.5 | 2.8 |
| 4 | A3F-YFP | 1.1±0.2 | 1.3 | 1.7±0.3 | 2.0 | 4.5^4^ | 1.1^4^ | 0.5 | 5.6^4^ |
| 5 | A3F-YFP | 1.2±0.4 | 1.9 | 1.8±0.6 | 3.0 | 1.0^4^ | 0.1^4^ | 3.8 | 1.1^4^ |
| 6 | A3F-YFP | 1.8±0.4 | 3.2 | 3.8±1.1 | 5.6 | 1.0^4^ | 2.9^4^ | 7.2 | 3.9^4^ |
| 7 | A3F-YFP | 1.1±0.6 | 1.9 | 2.0±0.7 | 2.9 | 1.0^4^ | 1.9^4^ | 0.6 | 2.9^4^ |
| 8 | A3F-YFP | - | - | - | - | 4.0 | 1.3 | - | 5.3 |
| 9 | A3F-YFP | - | - | - | - | 2.5 | 0.8 | - | 3.2 |
| 10 | A3F-YFP | - | - | - | - | 2.9 | 0.6 | - | 3.4 |
| 11 | IN-YFP | 0.9±0.4 | 1.3 | 1.4±0.8 | 2.2 | 3.1 | 5.3 | 0.3 | 8.4 |
| 12 | IN-YFP | 1.7±0.3 | 2.6 | 1.8±0.3 | 2.6 | 3.2 | 0.5 | 6.0 | 3.7 |
| 13 | IN-YFP | 2.3±0.7 | 3.2 | 4.3±1.0 | 5.5 | 7.3 | 2.1 | 0.9 | 9.4 |
| 14 | IN-YFP | 1.8±0.3 | 2.2 | 2.5±0.5 | 3.4 | 4.3 | 1.5 | 4.5 | 5.8 |
| 15 | IN-YFP | 1.8±0.4 | 2.2 | 2.5±0.5 | 3.2 | 7.1 | 1.4 | 1.8 | 8.5 |
| 16 | IN-YFP | 1.3±0.2 | 1.9 | 1.9±0.3 | 2.6 | 0.3 | 0.4 | 9.5 | 0.7 |
| 17 | IN-YFP | 1.5±0.4 | 1.9 | 2.1±0.7 | 3.1 | 2.9 | 1.4 | 5.7 | 4.2 |
| 18 | IN-YFP | 1.0±0.0 | 1.0 | 1.5±0.1 | 1.5 | 5.1 | 5.0 | 0.1 | 10.1 |
| 19 | IN-YFP | 1.8±0.3 | 2.2 | 2.2±0.3 | 2.5 | 4.2 | 0.6 | 1.9 | 4.8 |
| 20 | IN-YFP | 1.1±0.3 | 1.6 | 1.6±0.4 | 2.8 | 0.3 | 0.4 | 7.7 | 0.7 |
| 21 | IN-YFP | 1.4±0.2 | 1.9 | 1.5±0.3 | 2.0 | 2.8 | 1.4 | 5.4 | 4.2 |
| 22 | IN-YFP | 1.1±0.3 | 1.9 | 2.8±0.9 | 4.0 | 1.1 | 5.6 | 3.6 | 6.7 |
| 23 | IN-YFP | 1.2±0.4 | 1.9 | 2.6±0.8 | 3.9 | 1.9 | 1.8 | 4.1 | 3.7 |
| 24 | IN-YFP | 1.0±0.2 | 1.3 | 1.9±0.4 | 2.7 | 2.9 | 0.6 | 2.9 | 3.5 |
| 25 | IN-YFP | - | - | - | - | 0.6 | 0.6 | - | 1.2 |
| 26 | IN-YFP | - | - | - | - | 1.9 | 0.3 | - | 2.1 |
| 27 | IN-YFP | - | - | - | - | 1.4 | 1.2 | - | 2.6 |
| 28 | IN-YFP | - | - | - | - | 1.2 | 1.4 | - | 2.6 |
| 29 | IN-YFP | - | - | - | - | 0.3 | 0.4 | - | 0.7 |
| 30 | IN-YFP | - | - | - | - | 1.3 | 4.4 | - | 5.7 |
|  | **Avg. ± SD** | **1.4±0.4** | **2.1 ±0.6** | **2.3±0.8** | **3.2±1.1** | **2.8±1.9** | **1.5±1.6** | **3.8±2.7** | **4.3±2.6** |

^1^ A total of 21 HIV-1 complexes were automatically tracked after correction for nucleus movement (7 A3F-YFP labeled complexes [particles 1-7] and 14 IN-YFP labeled complexes [particles 11-24]), which are included in Figs. 3 and 4. Nine HIV-1 complexes were detected manually from additional movies (3 A3F-YFP labeled complexes [particles 8-10] and 6 IN-YFP complexes [particles 25-30]) to determine time in cytoplasm, NE residence time, and time of nuclear import.

^2^ No significant differences between the nuclear penetration distance, distance from point of nuclear entry, time in cytoplasm, NE residence time, observation time in nucleus, and time of nuclear import for A3F-YFP and IN-YFP complexes were observed (*P* > 0.05, *t*-test or Mann Whitney test); average values for both A3F-YFP and IN-YFP complexes are shown.

^3^ The time in cytoplasm represents the length of time between the time of infection and the time each viral complex arrived at the NE (see Fig 6A).

^4^ Four A3F-YFP labeled complexes were at the NE prior to beginning of movie (particles 4-7); therefore, the time in cytoplasm is an overestimate and the NE residence time is an underestimate for these complexes. The time in cytoplasm, NE residence time, and time of nuclear import for these particles was not included in the average values. The time in cytoplasm, NE residence time, and time of nuclear import for all particles except particles 4-7 (26 total) were included in Table 1 and Figs. 6 and 7.

^5^ Nuclear HIV-1 complexes were observed until the end of the movie or until they exited the z-stack.

^6^ hpi, hours post-infection.
